# Computational Neuroanatomy of Language: A Compartmental Framework From Cranial Landmarks to Subcortical Networks

**DOI:** 10.7759/cureus.113416

**Published:** 2026-07-26

**Authors:** Marco Obersnel, Kenneth X Probst, Chiara Angelini, Roberto Rodriguez Rubio

**Affiliations:** 1 Neurological Surgery, Catholic University of the Sacred Heart, Rome, ITA; 2 Neurological Surgery, University of California, San Francisco, San Francisco, USA; 3 Neurological Surgery, University of Ferrara, Ferrara, ITA; 4 Otolaryngology - Head and Neck Surgery, University of California, San Francisco, San Francisco, USA

**Keywords:** anatomy, awake surgery, cerebral cortex, craniometric points, dual-stream model, language, mapping, medical education, volumetric models, white matter

## Abstract

The anatomy underlying language functions has always had a pivotal role in brain surgery. Many language models have been developed over time, with the dual-stream model representing the most contemporary framework in a rapidly evolving field. However, the neurosurgical application of language models requires rigorous anatomical references and their systematic correlation with intraoperative language mapping, yet a comprehensive, layered anatomical-functional description integrating surgical relevance remains lacking. This study provides a structured, clinically applicable framework of language anatomy within a modern hodotopical perspective, integrating multilayered topographic descriptions with cadaveric dissections, tractographic reconstructions, and purpose-built anatomical illustrations. Both two-dimensional images and three-dimensional interactive models, generated through photogrammetry and structured-light scanning, are provided to facilitate spatial integration.

A focus on the relationship between craniometric points and underlying cortical and subcortical structures is presented with dedicated illustrations. The associative fibers involved in the computational neuroanatomy of language are organized into three compartments defined by the sylvian fissure as the principal anatomical landmark, distinguishing suprasylvian, infrasylvian, and retrosylvian. Within each compartment, white matter tracts are characterized as lateral or medial to the corona radiata and then described in three layers from superficial to deep.

Finally, a practical overview of the most common positive responses during awake language mapping is provided for each cortical and subcortical structure. Clinical intraoperative data here serve as the bridge between anatomical description and neuroscientific models.

This work is intended as a structured operative reference for neurosurgeons and trainees, fostering an integrated understanding of the three-dimensional computational anatomy of language-eloquent pathways.

## Introduction

Language perception and production rely on the coordinated activity of a complex network encompassing multiple cortical and subcortical structures [1]. Cortical regions involved in language are distributed bilaterally within and around the Sylvian fissure (SF), extending beyond the classical opercular areas to include the supplementary motor area (SMA) and perirolandic regions [2]. These regions are interconnected by subcortical white matter tracts, forming large-scale networks that are essential for language processing [2]. Over time, the classical Wernicke-Geschwind model has progressively given way to integrative, network-based accounts of language, including the dual-stream model and its subsequent refinements [3,4].

The dual-stream model conceptualizes language processing as supported by two partially parallel and interacting pathways. The dorsal stream is mainly involved in phonological processing and sensorimotor integration, linking auditory representations to articulatory-motor programs, whereas the ventral stream is mainly involved in semantic processing, linking auditory and lexical inputs to meaning [3]. In these frameworks, language is no longer attributed to isolated cortical centers but is understood as the result of distributed interactions within adaptable and densely connected cortico-subcortical networks. This perspective provides a more refined understanding of the distinct contributions of cortical and subcortical structures and their hierarchical organization [1,2]. Such a network-based view is consistent with the hodotopical perspective (from Greek ὁδός, hodos = path), in which brain functions arise from the dynamic interaction between cortical epicenters and the white matter pathways connecting them, rather than from a purely localizationist model in which each function is assigned to a single cortical area [2].

Recent advances in connectomics have further highlighted the role of broader cortical and subcortical networks in language processing, yet a comprehensive, layered anatomical-functional description of the dual-stream model remains lacking. This gap is of particular importance in neurosurgical practice, specifically brain surgery, where these pathways are frequently involved in intra-axial lesions. Surgical strategy must, therefore, integrate both detailed anatomical knowledge and intraoperative cortical and subcortical mapping.

This study provides a detailed, structured, and clinically applicable anatomical framework for neurosurgeons managing pathologies involving speech-related pathways. We aim to offer a practical, up-to-date resource for trainees and surgeons by presenting the "computational anatomy" of language, integrating multilayered topographic descriptions with their surgical relevance and contemporary connectomic functional implications. To facilitate the transition from traditional two-dimensional (2D) anatomy to a comprehensive three-dimensional (3D) perspective, we incorporate cadaveric dissections, dedicated diffusion magnetic resonance imaging (MRI)-based tractographic reconstructions, and purpose-built anatomical illustrations designed to enhance spatial integration. Finally, the implementation of anatomical volumetric models (VMs) and online 3D platforms provides additional visuospatial navigation of cortical and subcortical pathways, clarifying their complex relationships with surrounding intracranial structures.

Part of this work was presented at the "Comprehensive World Brain Mapping Practical Course" for educational purposes during the American Association of Neurological Surgeons 2024 Annual Scientific Meeting on May 3, 2024.

## Technical report

Materials and methods

Relevant cortical and subcortical brain anatomy was depicted using five brains (n = 10 hemispheres) obtained from cadaver donors through our institution's Willed Body Program, which prepared the hemispheres according to standard Klingler protocol [5]. The dissections were performed using a surgical microscope (OPMI Pentero 900; Zeiss, Oberkochen, Germany), and images were acquired with a high-definition camera (D850; Nikon, Tokyo, Japan) using standard macrophotography and modified ultraviolet reflectance techniques [6]. Specimen VMs were created using an image-based three-dimensional reconstruction workflow combining photogrammetry and structured-light scanning, as previously described.

To integrate 3D anatomical concepts, three base meshes (i.e., skull, brain, and ventricle) were acquired from our public 3D media repository, Sketchfab (Sketchfab Inc., New York). All meshes underwent further geometric postprocessing in Blender 4.4 (Blender Foundation, Amsterdam, the Netherlands) and Nomad Sculpt 2.7 (Hexanomad, Paris, France). Finally, photorealistic materials and textures were edited using Blender 4.4 (Blender Foundation, Amsterdam, the Netherlands). Institutional review board approval was not required for this anatomical study.

We combined high-resolution anatomical dissections of 10 human hemispheres with diffusion tractography to produce a multilayered lateral-to-medial description of the cortical and subcortical structures involved in language. Tractography of white matter tracts was performed on data from the Human Connectome Project (WU-Minn Consortium; PIs: Van Essen and Ugurbil; subject ID: 100307), using DSI Studio (Hou version; developed by Fang-Cheng Yeh, University of Pittsburgh, Pittsburgh, Pennsylvania) with generalized q-sampling imaging (GQI) reconstruction and orientation distribution function (ODF)-based deterministic fiber tracking.

All figures and interactive models, including the anatomical landmarks shown therein, were developed by the authors. Cadaveric images from the University of California, San Francisco Skull Base and Cerebrovascular Laboratory, as well as the original anatomical illustrations, were used with permission and annotated by the authors. Some of these images were presented for educational purposes during the "Comprehensive World Brain Mapping Practical Course" at the American Association of Neurological Surgeons 2024 Annual Scientific Meeting on May 3, 2024. All tables were created by the authors as original syntheses integrating the cited literature with original anatomical observations from the Skull Base and Cerebrovascular Laboratory, University of California, San Francisco.

Virtual platform

To improve interaction and navigation with relevant anatomical VMs obtained from dissections, model integrations, and tractographic reconstructions, the post-processed models were uploaded to a web-based 3D model viewer (Sketchfab, Sketchfab Inc., New York). The 3D scenes were optimized to allow for online real-time rendering, incorporating adjustments to position, lighting, materials, and filters. Pertinent anatomical structures were labeled and annotated for the interactive experience.

Superficial anatomy and layered organization

In this section, we present a multilayered anatomical description of the language network, beginning with superficial cranial landmarks and their relationship to underlying cortical structures. This framework then progresses into the deep white matter tracts, providing a cohesive map of the language-eloquent brain.

Craniometry: Correlation of Cranial and Cortico-Subcortical Structures

Precise knowledge of superficial landmarks and their three-dimensional relationships with underlying cortical and subcortical structures is fundamental to neurosurgical practice. While Pierre Paul Broca pioneered craniometry in 1876, correlating specific cranial points with cortical functions, this discipline remains the basis for modern "tailored" craniotomies and precise cortical localization [7].

**Figure 1:**
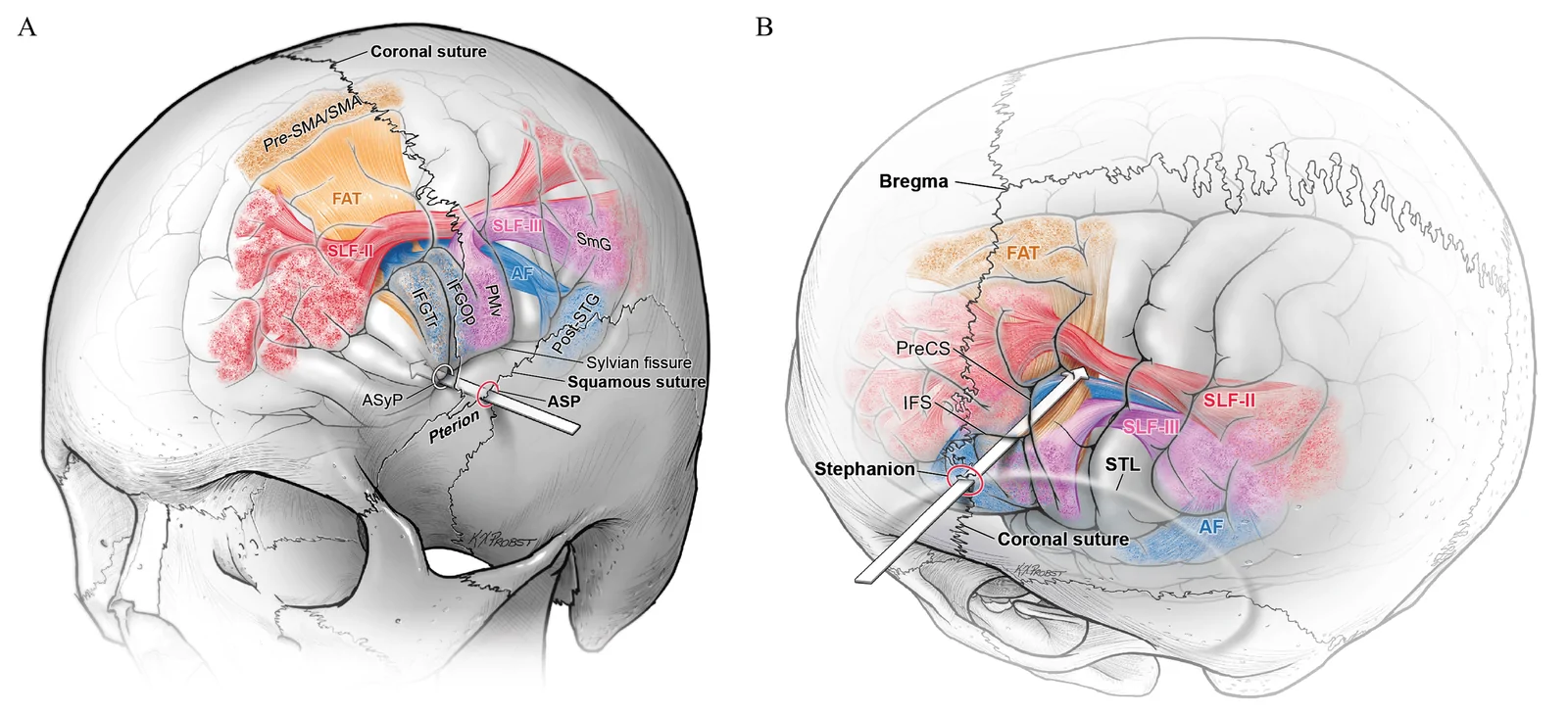
Craniometric landmarks of the lateral skull and their cortical and subcortical relationships within the suprasylvian compartment. (A) Anterolateral view of the skull illustrating the pterion and ASP in relation to their underlying structures. The pterion lies directly over the Sylvian fissure, while the ASP corresponds to the ASyP. Deep to this region lies the frontal operculum, including the IFGTr and IFGOp, as well as the PMv. Subcortically, this area represents a convergence zone of major association pathways, where the frontal terminations of the FAT, AF, and SLF III can be identified. The FAT ascends medially toward the SMA complex. The SLF II courses more superficially than the FAT, curving around it and bending inferiorly after crossing it. Posteriorly, additional SLF III terminations are observed in the supramarginal gyrus within the retrosylvian compartment, while the temporal terminations of the AF extend into the infrasylvian compartment. (B) Posterolateral view of the skull illustrating the bregma and stephanion in relation to their underlying structures. The stephanion corresponds to the region just anterior to the precentral gyrus, at the junction between the inferior frontal sulcus and the precentral sulcus, delimiting the superior and posterior margin of the inferior frontal gyrus. Deep to this area lies the premotor cortex and adjacent frontal regions. Subcortically, this region represents a key intersection of major association pathways, including the SLF II, SLF III, and AF, in close relationship with the FAT. In particular, SLF II intersects with the FAT at this level, while SLF III and AF course more ventrally. At the level of the bregma, the underlying medial frontal cortex includes the supplementary motor area (SMA complex), which represents a medial termination of the FAT. Courtesy of the Skull Base and Cerebrovascular Laboratory, University of California, San Francisco; reproduced with permission. AF, arcuate fasciculus; ASP, anterior squamous point; ASyP, anterior Sylvian point; FAT, frontal aslant tract; IFGOp, pars opercularis of the inferior frontal gyrus; IFGTr, pars triangularis of the inferior frontal gyrus; IFS: inferior frontal sulcus; PMv, ventral premotor cortex; Post-STG: posterior superior temporal gyrus; PreCS: precentral sulcus; SLF II, superior longitudinal fasciculus II; SLF III, superior longitudinal fasciculus III; SMA, supplementary motor area; SmG: supramarginal gyrus; STL: superior temporal line.

**Figure 2:**
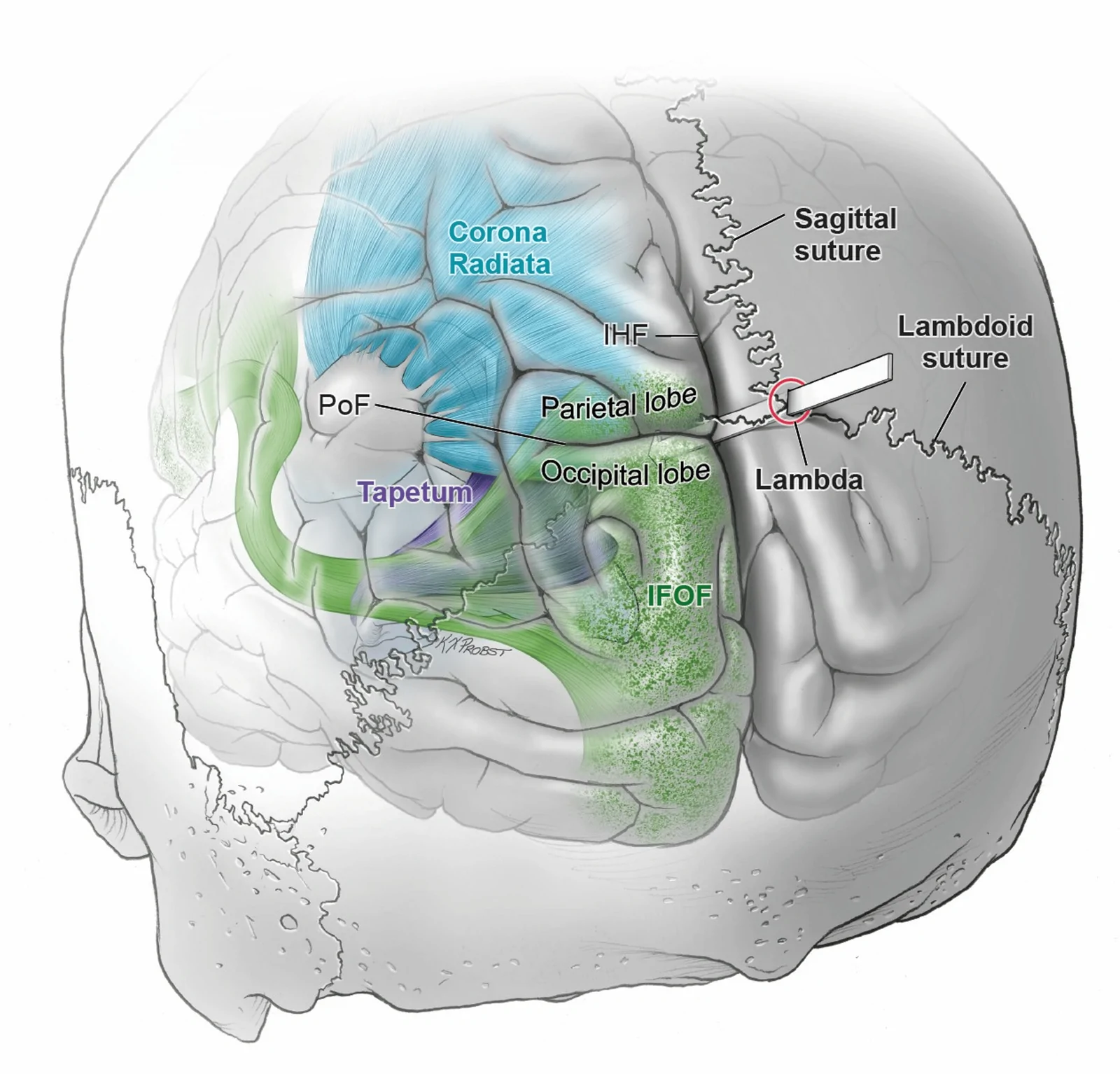
Lateral-posterior view of the skull illustrating the lambda in relation to its underlying structures, focusing on the retrosylvian compartment. The lambda corresponds to the junction of the sagittal and lambdoid sutures and overlies the parieto-occipital region. Cortically, this point is located superior to the PoF, which lies inferior and lateral to it and corresponding to the posterior aspect of the precuneus along the IHF. The surrounding cortex includes the posterior parietal and occipital lobes. Subcortically, this region corresponds to the transition of the corona radiata into the tapetum, forming part of the sagittal stratum. At this level, the deep white matter is organized in layered structures, including the tapetum and long association pathways such as the inferior IFOF, which courses within the temporo-parieto-occipital region. Courtesy of the Skull Base and Cerebrovascular Laboratory, University of California, San Francisco; reproduced with permission. IFOF, inferior fronto-occipital fasciculus; IHF, interhemispheric fissure; PoF, parieto-occipital fissure.

**Figure 3:**
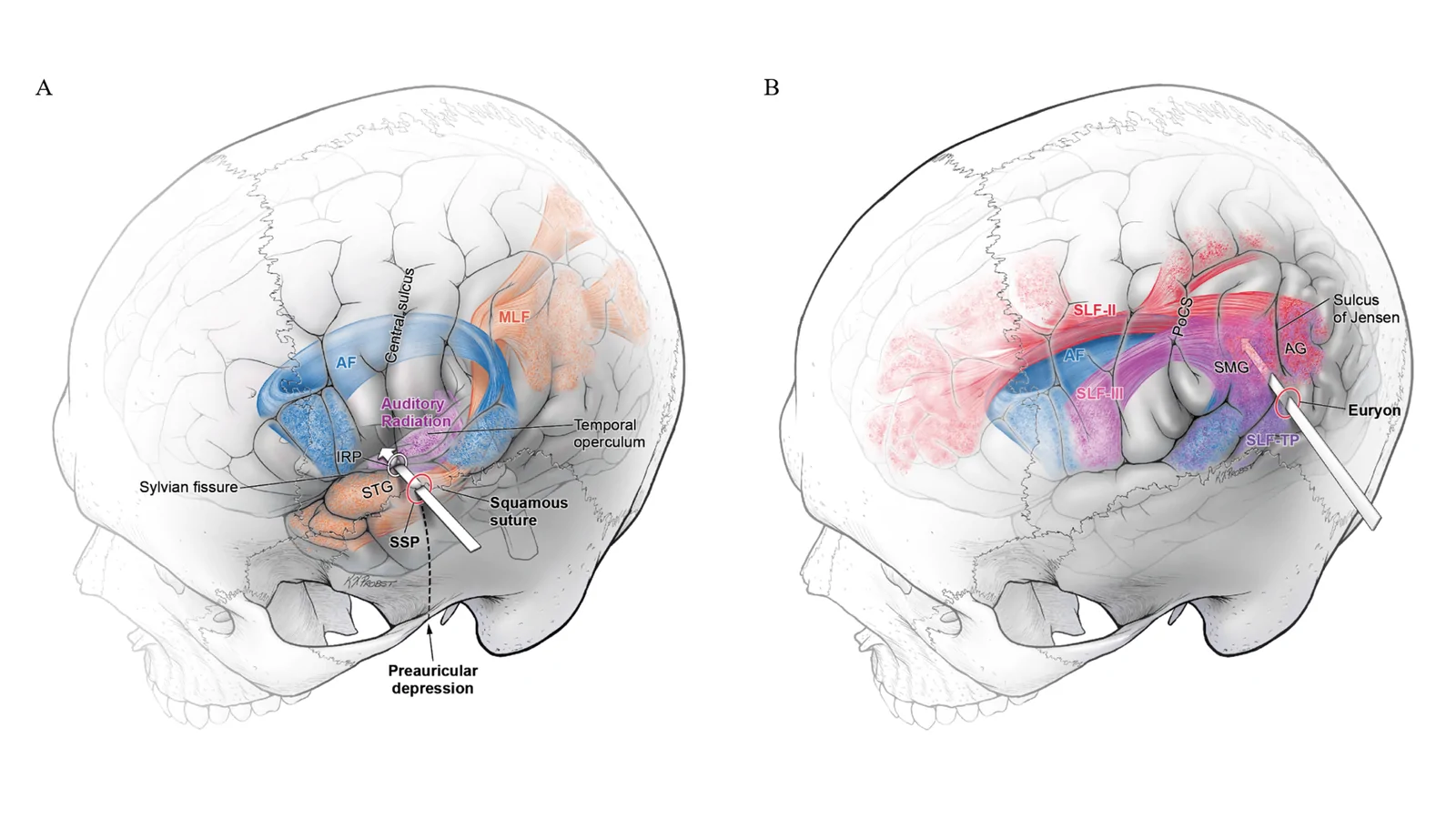
Lateral views of the skull illustrating two key surface landmarks of the infrasylvian and retrosylvian compartments. (A) The SSP lies on the squamous suture and is vertically aligned with the preauricular depression. This landmark corresponds topographically to the IRP, located at the intersection between the central sulcus and the Sylvian fissure. Cortically, this area includes the superior temporal gyrus and Heschl’s gyrus. Subcortically, it corresponds to the AF, the MLF, and the auditory radiations. (B) The euryon corresponds to the parietal tuberosity and lies posterior to the postcentral sulcus, overlying the inferior parietal lobule, including both the SMG and the AG. It is located lateral to the intraparietal sulcus and anterior to the sulcus of Jensen. This region corresponds to the so-called Geschwind's territory. Subcortically, this area overlies the AF beneath the SMG, the convergence zone of the SLF III and the SLF-TP, and in depth, the anterior portion of the sagittal stratum, including the tapetum, optic radiations, ILF, MLF, and IFOF. Courtesy of the Skull Base and Cerebrovascular Laboratory, University of California, San Francisco; reproduced with permission. AF, arcuate fasciculus; AG, angular gyrus; IFOF, inferior fronto-occipital fasciculus; ILF, inferior longitudinal fasciculus; IRP, inferior rolandic point; MLF, middle longitudinal fasciculus; SLF III, superior longitudinal fasciculus III; SLF-TP, temporoparietal component of the superior longitudinal fasciculus; SMG, supramarginal gyrus; SSP, superior squamous point; STG, superior temporal gyrus.

Despite the prevalence of advanced neuronavigation, craniometric expertise is indispensable during surgical planning and intraoperative navigation. It serves as a vital safeguard when the reliability of image-guided systems is compromised by brain shift or limited surgical access. To facilitate this multilayered conceptualization, Table 1 summarizes the most relevant superficial landmarks and their direct correlation to the underlying cortical and subcortical structures within the perisylvian network [7]. Figures 1-3 provide a three-dimensional representation of these structures from superficial to deep.

**Table 1:** Craniometric points and pertinent cortical-subcortical relationships. AC, anterior commissure; AF, arcuate fasciculus; AG, angular gyrus; ASP, anterior squamous point; ASyP, anterior Sylvian point; Br, bregma; CC, corpus callosum; CS, central sulcus; CST, corticospinal tract; Eu, euryon; FAT, frontal aslant tract; FST, frontostriatal tract; IFG, inferior frontal gyrus; IFS, inferior frontal sulcus; IHF, interhemispheric fissure; IFOF, inferior fronto-occipital fasciculus; ILF, inferior longitudinal fasciculus; IPP, intraparietal point; IPS, intraparietal sulcus; IRP, inferior rolandic point; Lm, lambda; MLF, middle longitudinal fasciculus; Na, nasion; PC, posterior commissure; PCoP, posterior coronal point; PoCS, postcentral sulcus; PostSTS, posterior superior temporal sulcus; PreCG, precentral gyrus; PreCS, precentral sulcus; PSyP, posterior Sylvian point; Pt, pterion; PTP, posterior temporal point; SF, Sylvian fissure; SFS, superior frontal sulcus; SLF I, superior longitudinal fasciculus I; SLF II, superior longitudinal fasciculus II; SLF III, superior longitudinal fasciculus III; SLF-TP, temporoparietal component of the superior longitudinal fasciculus; SMA, supplementary motor area; SMG, supramarginal gyrus; SRP, superior rolandic point; SSP, superior squamous point; St, stephanion; STG, superior temporal gyrus; STL, superior temporal line; STS, superior temporal sulcus. Sources: Reference [7].

Craniometric point	Location	Cortical relationship(s)	Subcortical relationship(s)
Bregma (Br)	Junction of the coronal and sagittal sutures.	The PreCG lies approximately 4.5 cm posterior to Br. More medially, the SRP, defined by the junction of the CS with the IHF, is located along the sagittal suture about 5 cm posterior to Br. Br is about 25 mm anterior to the line passing through AC and perpendicular to the AC-PC line, about 5 mm anterior to the anterior margin of the SMA.	SRP is close to the primary motor cortex, where the CST begins. SRP is also approximately aligned, in the coronal plane, with the quadrigeminal cistern and the splenium of the corpus callosum. SMA is a termination of FAT and FST.
Posterior coronal point (PCoP)	1.5 cm posterior to Br and 3 cm lateral to the sagittal suture.	SFS/PreCS point: junction between the SFS and PreCS; landmark for the omega region (hand motor activation area).	The SFS/PreCS point is at the same coronal plane as the thalamus and the inferior aspect of the lateral ventricle, just behind the foramen of Monro. Underneath lie the thalamic radiations and internal capsule.
Lambda (Lm)	Junction of the lambdoid and sagittal sutures.	The parieto-occipital fissure (where the parieto-occipital sulcus emerges inside the IHF) lies inferior and lateral to Lm, a landmark corresponding to the posterior aspect of the precuneus along the IHF.	Transition of the corona radiata into the tapetum.
Obelion	Intersection of the sagittal suture with the line connecting the two foramina of the parietal emissary veins, 2.5 cm anterior to Lm.	Superior parietal lobule; posterior portion of precuneus.	Precuneus is the parietal termination of SLF I.
Intraparietal point (IPP)	6 cm anterior to Lm and 5 cm lateral to the sagittal suture.	IPS/PoCS point: connection between the IPS and the post-central gyrus.	Coronal projection of the IPS/PoCS point corresponds to the sagittal stratum (tapetum, auditory and optic radiation, ILF, MLF, and IFOF) and then the atrium of the lateral ventricle.
Pterion (Pt)	H-shaped sutural junction between the frontal, parietal, temporal, and sphenoid bones.	Lies directly above the SF.	Posterior STG is a termination of AF and MLF and has connections with ILF and IFOF.
Superior squamous point (SSP)	Intersection of the highest portion of the squamous suture with a vertical line that ascends from the preauricular depression.	IRP is located at the intersection between the SF and the CS. Underneath lies the posterior aspect of the STG (Wernicke's area) and Heschl's gyrus.	Temporal portion of AF; MLF; auditory radiations.
Anterior squamous point (ASP)	Posterior portion of the pterion, 2–2.5 cm anterior to the SSP.	Underneath lies the ASyP, which is inferior to pars triangularis, immediately anterior to pars opercularis (Broca's area).	Pars opercularis of the IFG is a termination of AF, SLF III, and FAT.
Posterior temporal point (PTP)	3 cm above the ascending line from the meeting point between the parietomastoid and the squamosal sutures.	PostSTS: posterior segment of the STS, where its central, predominantly horizontal branch enters the angular gyrus. PSyP: 2–3 cm anterosuperior to the PostSTS (anterior to the bifurcation of the STS).	Its axial projection corresponds to the sagittal stratum (tapetum, auditory and optic radiations, ILF, MLF, and IFOF) and then the atrium of the lateral ventricle.
Stephanion (St)	Intersection between the STL and the coronal suture. Roughly 8 cm lateral to Br.	IFS/PreCS point (meeting point of the IFS and PreCS): approximately 0.5 cm posterior to St. It identifies the PreCG and delimits the superior and posterior margin of the IFG. STL marks the superior limit of the IFG.	SLF II intersection with FAT; SLF III; AF.
Euryon (Eu)	Palpable prominence in the middle of the parietal tuberosity.	Always behind the PoCS. Landmark for the inferior parietal lobule, in particular, the posterior aspect of the SMG next to the AG. Located 1–3 cm lateral to the IPS and 1–2 cm anterior to the sulcus of Jensen.	Underneath the SMG passes the AF. SMG is also the convergence point of SLF III and SLF-TP. Slightly above passes the SLF II. Geschwind's area: anterior part of sagittal stratum (tapetum, auditory and optic radiation, ILF, MLF, and IFOF).
Nasion (Na)	Midpoint of the frontonasal suture, at its intersection with the nasal suture. Visible as a midline depression superior to the nose bridge and between the two eyes.	Frontal pole.	Aligned, in the axial plane, with the CC, approximately 12.5 cm anterior to the genu and 20 cm anterior to the splenium.

Cortical Structures

The relationship between neural structures and their functions was first described by Pierre Paul Broca and John Hughlings Jackson around 1870. Fedor Krause produced the first human cortical map in 1911, followed by Wilder Penfield’s detailed map in 1937, which characterized motor, sensory, and language areas [8]. Language-related cortical regions are mainly located in the Sylvian and perisylvian areas, with additional contributions from the SMA complex and premotor cortex.

The Sylvian Fissure

**Figure 4:**
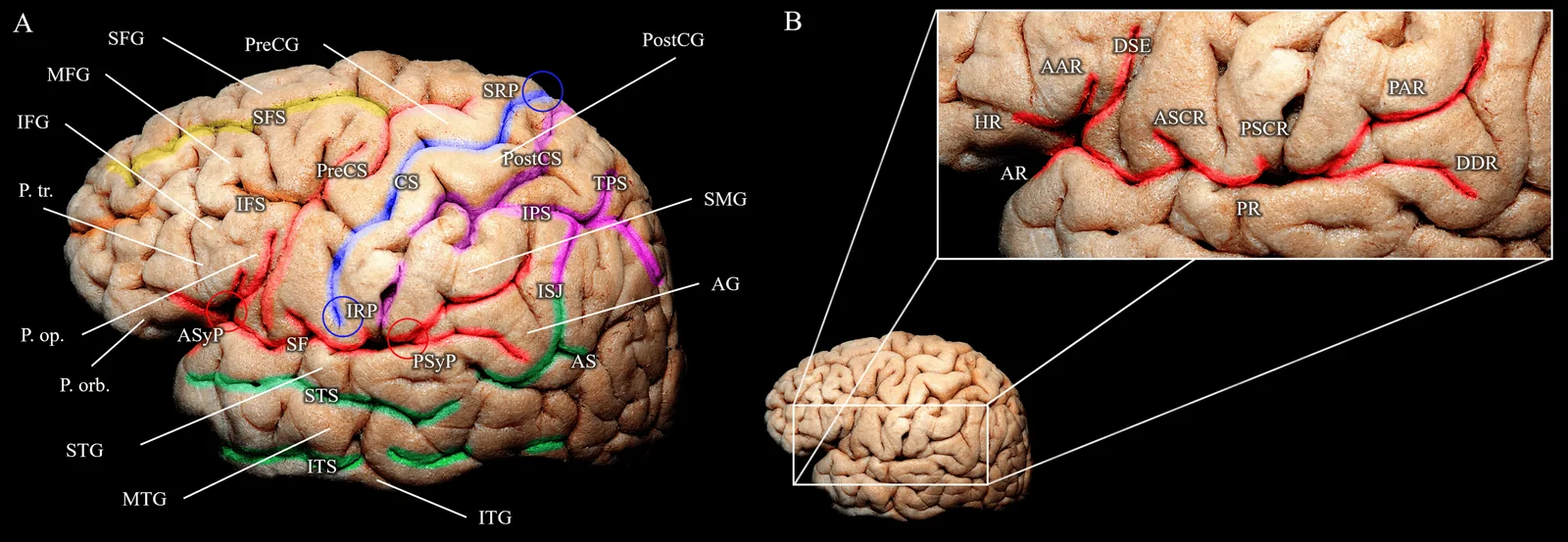
Lateral view of the cortex of the left hemisphere. (A) The main cortical gyri and sulci are depicted. The SFG, MFG, and IFG are identified, the latter subdivided into the P. op., P. tr., and P. orb. The SFS and IFS are shown. The PreCG and PostCG are separated by the CS (blue), whose SRP and IRP are identified; the PreCS and PostCS are also visible. The IPS (purple) extends posteriorly toward the parietal region and gives rise to two vertical branches: a smaller superior fold, the TPS, and the ISJ, which separates the SMG from the AG. The SF (red) and its ASyP and PSyP are highlighted. The STG, MTG, and ITG are delineated by the STS and ITS. Posteriorly, the STS bifurcates into an ascending branch, corresponding to the ISJ, and a horizontal branch, the AS, which courses into the AG. (B) Magnified view of the perisylvian region illustrating the organization of the SF and its main branches. The SF is composed of an AR and a PR, with the ASyP marking their transition. From the anterior portion arise the HR and AAR, which separate the P. orb., P. tr., and P. op. of the IFG. Posterior to the AAR, the DSE is visible, dividing the anterior basal portion of the P. op. The PR courses obliquely backward and upward toward the inferior parietal lobule. Along its course, smaller branches arise, including the ASCR and PSCR, which appear as short ascending rami. More distally, the PR bifurcates into the PAR and DDR, directed respectively toward the SMG and STG. Courtesy of the Skull Base and Cerebrovascular Laboratory, University of California, San Francisco; reproduced with permission. AAR, anterior ascending ramus; AG, angular gyrus; AR, anterior ramus; AS, angular sulcus; ASCR, anterior subcentral ramus of the Sylvian fissure; ASyP, anterior Sylvian point; CS, central sulcus; DDR, descending ramus; DSE, diagonal sulcus of Eberstaller; HR, horizontal ramus; IFG, inferior frontal gyrus; IFS, inferior frontal sulcus; IPS, intraparietal sulcus; IRP, inferior Rolandic point; ISJ, intermediate sulcus of Jensen; ITG, inferior temporal gyrus; ITS, inferior temporal sulcus; MFG, middle frontal gyrus; MTG, middle temporal gyrus; P. op., pars opercularis; P. orb., pars orbitalis; P. tr., pars triangularis; PAR, posterior ascending ramus; PostCG, postcentral gyrus; PostCS, postcentral sulcus; PR, posterior ramus; PreCG, precentral gyrus; PreCS, precentral sulcus; PSCR, posterior subcentral ramus of the Sylvian fissure; PSyP, posterior Sylvian point; SF, Sylvian fissure; SFG, superior frontal gyrus; SFS, superior frontal sulcus; SMG, supramarginal gyrus; SRP, superior Rolandic point; STG, superior temporal gyrus; STS, superior temporal sulcus; TPS, transverse parietal sulcus of Brissaud.

The Sylvian and perisylvian regions comprise cortical structures located in the frontal, temporal, and parietal lobes. They are defined by the lateral segment of the SF, which demarcates the temporal lobe inferiorly from the frontal and parietal lobes superiorly (Figure 4). These structures are highly interconnected, forming a perisylvian network in which every area contributes to language processing. In particular, the posterior temporal lobe, inferior parietal lobule, and inferior frontal gyrus (IFG) are considered key players.

Its approximate depth typically ranges from 1 to 3 cm, and it is one of the continuous sulci of the brain. The lateral SF is formed by a deep anterior branch and a posterior branch containing the Sylvian cistern. Its lateral portion consists of two anterior rami, horizontal and ascending, and, commonly, a single or double posterior ramus. The anterior horizontal ramus demarcates the pars orbitalis and triangularis of the IFG; the anterior ascending ramus separates the pars triangularis and opercularis of the IFG; and the posterior ramus or rami divide the parietal and temporal lobes, coursing posterosuperiorly toward the inferior parietal lobule.

In addition, the diagonal sulcus of Eberstaller (DSE), another branch of SF, can be found behind the anterior ascending ramus, dividing the anterior basal portion of the pars opercularis, and the anterior and posterior subcentral rami can be located as little ascending rami from the posterior branch of SF.

The anterior Sylvian point (ASyP) is a slight widening of the subarachnoid space from which the horizontal and ascending anterior rami typically emerge. It separates the proximal from the distal portions of the SF, and it is located below the pars triangularis and in front of the base of the pars opercularis of the IFG. Near the posterior ramus, another widening of the SF, the posterior Sylvian point (PSyP), can be identified at the origin of two additional rami. The posterior ascending branch terminates within the supramarginal gyrus, whereas a distal descending branch reaches the STG.

The Temporal Lobe

The posterior portion of the superior temporal gyrus (STG) is included between the SF and the superior temporal sulcus (STS). Posteriorly, the STS, in a similar fashion as the posterior portion of the SF, bifurcates into an ascending segment, also called the intermediate sulcus of Jensen (ISJ, sometimes considered an offshoot of the intraparietal sulcus), which forms the boundary between the supramarginal gyrus and the angular gyrus (AG), and a horizontal segment known as the angular sulcus (AS), which penetrates the AG.

The posterior STG forms the temporal operculum, covering the inferior surface of the insula. Its superior, or opercular, surface includes transverse gyri that project obliquely toward the inferior part of the circular insular sulcus. The transverse gyrus of Heschl is the most prominent of these: it originates in the posterior portion of the STG and follows an oblique trajectory toward the posterior apex of the SF floor and the ventricular atrium. This exhibits high morphological variability across individuals and brain hemispheres, including the presence of a complete or partial sulcus intermedius and, consequently, a full or partial duplication of Heschl’s gyrus. Primary auditory cortex seems to be located in the whole Heschl’s gyrus, whether single or duplicated, and sometimes it continues to extend onto less pronounced gyri of the planum temporale. The tonotopic gradient runs parallel to the long axis of the gyrus, and frequency representation has a “high-low-low-high” cortical distribution.

The STS is always sharply defined and deep, and it usually appears as a continuous sulcus parallel to the SF. In contrast, the inferior temporal sulcus (ITS) is generally interrupted and composed of several segments.

The posterior middle temporal gyrus (MTG), located between the STS and ITS, often shows continuity with the AG deep to the distal horizontal segment of the STS and usually extends inferiorly toward the inferior occipital gyrus.

Posteriorly, the inferior temporal gyrus (ITG) is continuous with the inferior occipital gyrus across the preoccipital notch, a landmark that defines the posterior boundary of the temporal lobe. Inferiorly, it runs along the inferolateral edge of the cerebral hemisphere. On its medial basal surface, the ITG is adjacent to the lateral temporooccipital, or fusiform, gyrus and to the temporooccipital sulcus, which lies between the two gyri.

According to the dual stream model, many of these posterior temporal cortical structures are bilaterally involved in language processing. In contrast, some anterior temporal, parietal, and frontal regions exhibit hemispheric lateralization, especially in the dominant hemisphere.

The anterior MTG is generally shorter, bringing the STG and the ITG into continuity and thereby forming the temporal pole.

The Parietal Lobe

The STG continues posteriorly and superiorly to the supramarginal gyrus (SMG), which, with its curved shape, surrounds the posterior ascending branch of the SF. It connects anteriorly to the postcentral gyrus through a cortical bridge around the inferior postcentral sulcus, and posteriorly sometimes wraps around the intermediate sulcus of Jensen toward the AG.

The AG extends around the distal horizontal branch of the STS, or angular sulcus, and connects anteriorly to the MTG. Posteriorly, it gives rise to a fold that connects it to the middle occipital gyrus. Together, the SMG and AG form the inferior parietal lobule, also referred to as Geschwind’s territory. This region is bounded superiorly by the intraparietal sulcus, which has a gently arched course with inferior concavity and usually joins the inferior portion of the postcentral sulcus anteriorly while becoming the intraoccipital sulcus posteriorly. The intraparietal sulcus usually has two vertical branches: a smaller superior fold, the transverse parietal sulcus of Brissaud, and a more developed inferior fold, corresponding to the previously described sulcus of Jensen, that divides SMG and AG.

The Frontal Lobe

The IFG is irregular in shape; it is delineated and crisscrossed inferiorly and superiorly by branches of, respectively, the SF and the inferior frontal sulcus (IFS). Anteriorly, it terminates by joining the anterior portion of the middle frontal gyrus (MFG); posteriorly, it communicates with the precentral gyrus. The IFG consists, in an anteroposterior sequence, of its pars orbitalis, triangularis, and opercularis.

Pars orbitalis is the most prominent of the three. Inferiorly, it continues to the lateral orbital gyrus, occasionally passing deep to a shallow sulcal indentation known as the frontoorbital sulcus.

Pars triangularis is characterized by the emergence of the horizontal and ascending anterior rami from the ASyP. Pars triangularis is frequently subdivided superiorly by a small descending branch of the IFS, and it appears more recessed than the other two pars.

Pars opercularis has a U-shaped configuration, and it is always surrounded by the precentral sulcus. It is delineated anteriorly and inferiorly by the ASyP and posteriorly by the anterior subcentral ramus of the SF. Sometimes, the anterior basal portion of the opercular part shows greater development and is separated by the diagonal sulcus of Eberstaller.

Pars opercularis, together with the anterior segment of the inferior precentral gyrus, forms the ventral premotor cortex (vPMC). The posterior portion of the inferior precentral gyrus is the primary motor cortex of muscles related to speech, including the tongue, lips, and pharynx [5].

The frontoparietal operculum is formed by the pars triangularis and opercularis, the subcentral gyrus (which bridges the precentral and postcentral gyri), and the anterior inferior SMG. It covers the superior insular surface, located between the horizontal and posterior ascending SF branches.

Finally, the posterior MFG, also known as the dorsolateral premotor cortex (dlPMC), lies between the superior frontal sulcus (SFS) and the IFS. Usually, there is an interruption of the IFG that connects the middle and inferior frontal gyri. In the majority of human hemispheres, a prominent cortical bridge links the MFG to the precentral gyrus at the site of a clear gap in the precentral sulcus [8].

The SMA Complex

**Figure 5:**
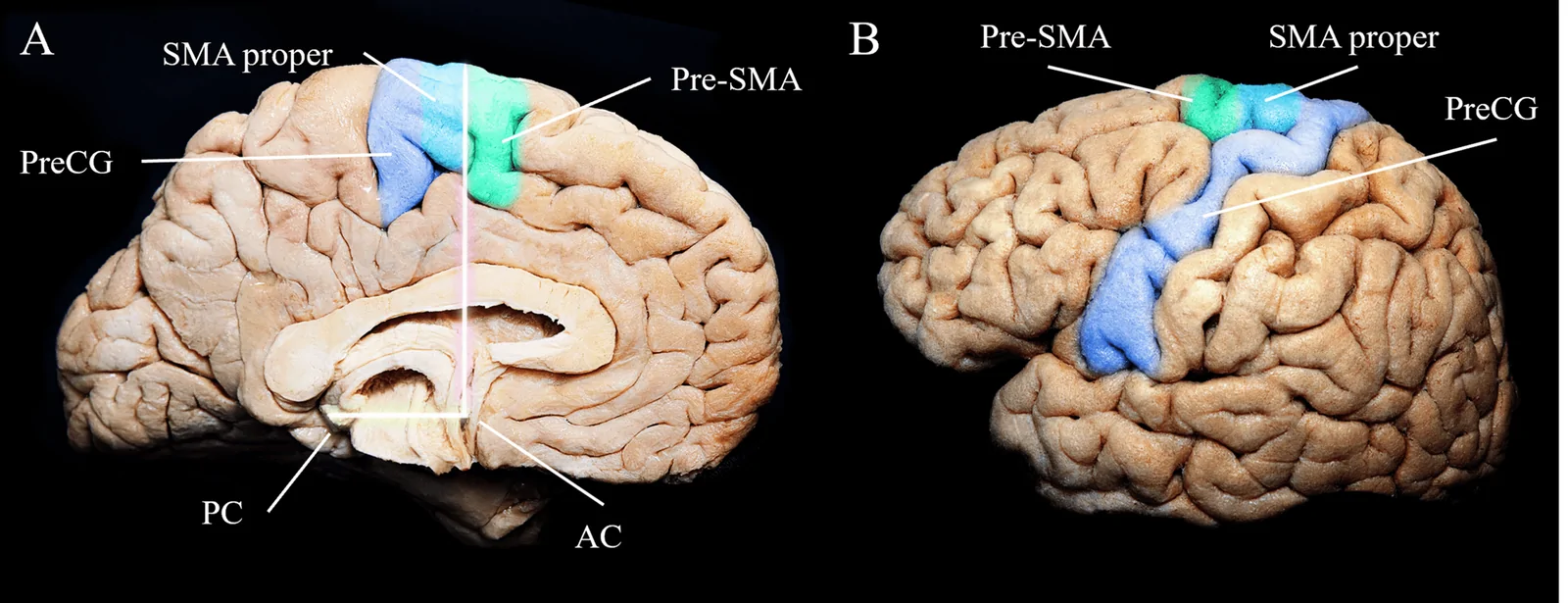
Medial and lateral views of the left hemisphere highlighting the SMA. (A) Medial view. The SMA proper (blue) and pre-SMA (green) are shown anterior to the PreCG. The boundary between the pre-SMA and SMA proper is defined by a vertical line crossing the AC and drawn perpendicular to the AC–PC line. The AC and PC are shown as deep anatomical landmarks. (B) Lateral view. The SMA proper (blue) extends onto the superior frontal gyrus and reaches the lateral convexity, where it lies immediately anterior to the PreCG (light blue). The pre-SMA (green) is located anteriorly. Courtesy of the Skull Base and Cerebrovascular Laboratory, University of California, San Francisco; reproduced with permission. AC, anterior commissure; PC, posterior commissure; PreCG, precentral gyrus; SMA, supplementary motor area.

The SMA complex is located on the dorsomedial surface of the superior frontal gyrus (SFG), anterior to the precentral gyrus. Medially, it is bordered by the cingulate sulcus; laterally, the SFS is a common surgical landmark, although following functional studies, the lateral border should be located 15 mm from the midline (Figure 5). The anterior border is challenging to define anatomically. Still, a rostral cytoarchitectonic limit can be described 20 mm anterior to the vertical line that transverses the anterior commissure and is perpendicular to the line between the anterior and posterior commissures (AC-PC line).

This area can be divided into two components, the pre-SMA (anteriorly) and the SMA proper (posteriorly), by drawing the vertical line crossing the level of the anterior commissure and oriented perpendicular to the AC-PC line [9].

Subcortical Structures

Several methods have been proposed to harden brain tissue for white matter dissection, from alcohol immersion to deep frying. In 1935, Josef Klingler introduced a refined technique: freezing formalin-fixed brains, which crystallizes formalin and mechanically separates fibers, significantly improving tract visibility and dissection [5]. The classification of white matter fibers, distinguishing association, commissural, and projection tracts, was initially defined by Theodor Meynert in 1867 [9].

Association fibers connect cortical regions within the same hemisphere (short fibers between adjacent gyri and long fibers between distant areas); commissural fibers link homologous regions across hemispheres; and projection fibers link the cerebral cortex to subcortical nuclei, brainstem, cerebellum, and spinal cord. These systems are studied through dissection, voxel-based imaging (DTI), and direct electrical stimulation (DES), the current gold standard; transcranial magnetic stimulation (TMS) offers a non-invasive alternative.

Cortical and subcortical structures are inherently three-dimensional and show marked inter- and intra-individual variability, influenced by age, pathology, and neuroplasticity. In low-grade gliomas, functional reorganization can even allow delayed surgical intervention.

White matter must be viewed as a dynamic network within a hodotopical framework, where overlapping networks support motor, language, cognitive, and emotional functions. Modern neuroanatomy is therefore best understood as computational neuroanatomy, integrating structure, function, and adaptability.

**Figure 6:**
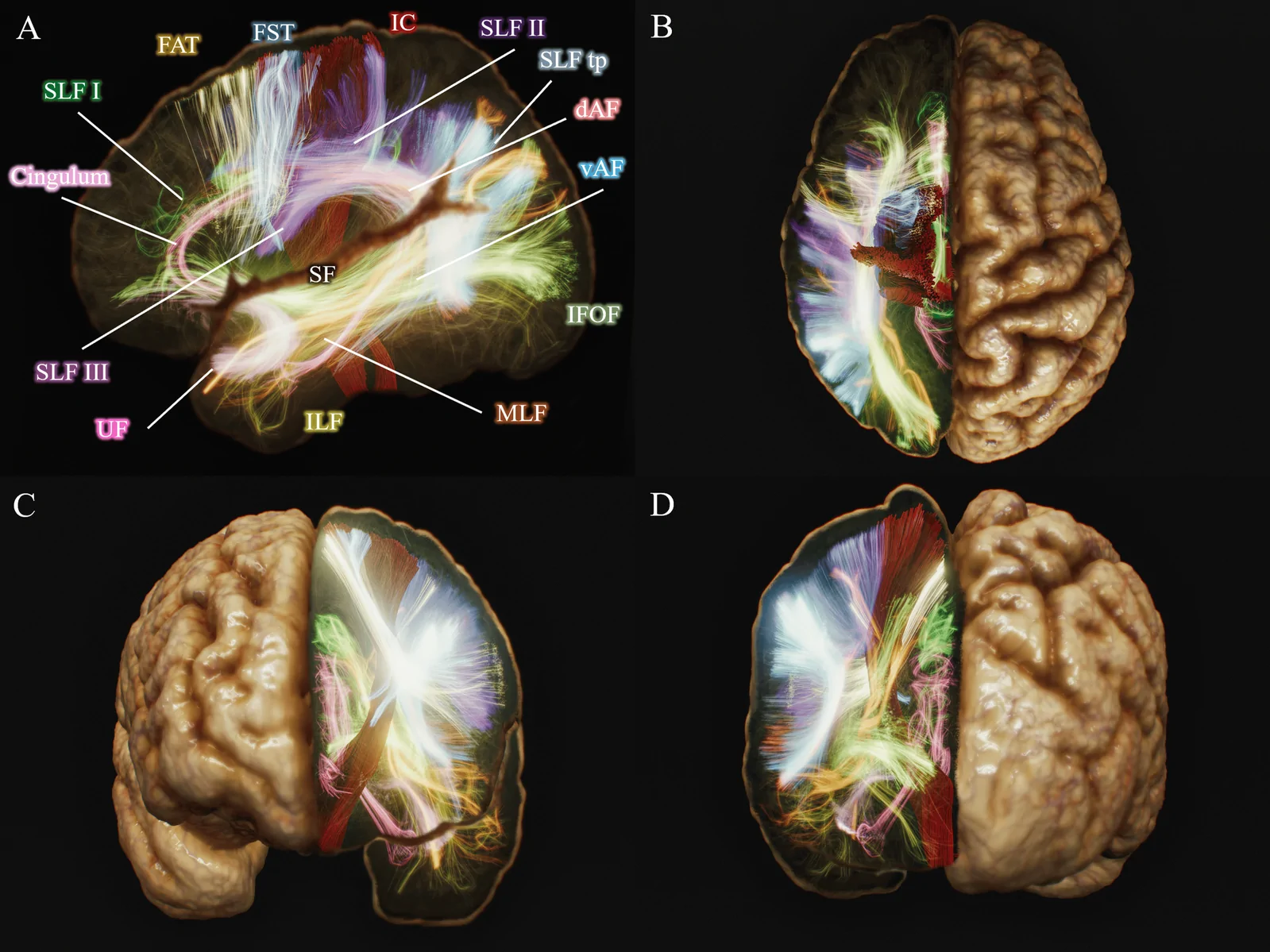
Spatial organization of speech-related white matter tracts according to their relationship with the SF. (A) Dorsolateral view of the left hemisphere showing the main association fibers. The SF serves as a key anatomical landmark distinguishing three compartments: suprasylvian, infrasylvian, and retrosylvian. In the suprasylvian compartment, the SLF I, SLF II, SLF III, FAT, and FST are identified. In the infrasylvian compartment, the ILF, UF, IFOF, and MLF are shown. The AF, including its dAF and vAF components, courses from the suprasylvian region, arches through the retrosylvian compartment, and extends into the infrasylvian region. Retrosylvian fibers include the SLF-tp, together with the MLF, IFOF, and ILF. The cingulum and SLF I are visualized medially. The IC is highlighted in red. (B) Superior view illustrating the topographical arrangement of white matter tracts around the IC (red). Medially, the SLF I (green) and cingulum (pink) are identified along the interhemispheric region. Immediately lateral to the IC, the FAT (light orange) and FST (light blue) are appreciable. Anterolaterally, the SLF II (purple) and SLF III (violet) are identified. In the retrosylvian region, the SLF-tp (light blue) is seen laterally, whereas the IFOF (light green) and MLF (orange) are positioned more medially. (C) Anterior view highlighting the lateral organization of white matter tracts around the IC (red). Medially, the SLF I (green) and cingulum (pink) are identified. The FAT (light orange) and FST (light blue) are appreciable, arising from the supplementary motor area: the FAT projects laterally, whereas the FST curves medially toward the basal ganglia. In the suprasylvian region, the SLF II (purple) and SLF III (violet) are identified together with the AF (dAF, dark orange; vAF, blue), whose anterior terminations toward the frontal operculum are appreciable; frontal fibers of the IFOF (light green) can also be partially appreciated. In the infrasylvian region, the ILF (yellow) and MLF (orange) fibers are visible within the temporal lobe, coursing inferiorly and superiorly, respectively. (D) Posterior view highlighting the retrosylvian organization of white matter tracts. The IC (red) is identified. Medially, the cingulum (pink) and SLF I (green) are visible. Superiorly, the SLF II (purple) is appreciable. Laterally, the SLF-tp (light blue) is identified. The AF, with its dAF (dark orange) and vAF (dark blue), arches posteriorly toward the temporoparietal junction. More medially, the IFOF (light green) and MLF (orange) are identified, with the MLF projecting posteriorly toward the superior parietal lobule, cuneus, precuneus, and lateral occipital cortex. Inferiorly, the ILF (yellow) is appreciable within the temporal lobe. Courtesy of the Skull Base and Cerebrovascular Laboratory, University of California, San Francisco; reproduced with permission. AF, arcuate fasciculus; dAF, dorsal arcuate fasciculus; FAT, frontal aslant tract; FST, frontostriatal tract; IC, internal capsule; IFOF, inferior fronto-occipital fasciculus; ILF, inferior longitudinal fasciculus; MLF, middle longitudinal fasciculus; SF, Sylvian fissure; SLF I–III, superior longitudinal fasciculus I–III; SLF-tp, temporoparietal component of the superior longitudinal fasciculus; UF, uncinate fasciculus; vAF, ventral arcuate fasciculus.

To translate this conceptual framework into surgical practice, we propose a spatial ontology organized around nested principles (Table 2, Figure 6, Interactive Model 1).

**Table 2:** Classification of the white matter tracts relevant for speech function according to their spatial organization relative to two sets of reference structures: the sylvian fissure and its compartments in the sagittal plane, and the corona radiata, internal capsule, and inferior thalamic peduncle in the coronal plane. ^†^Dorsal fibers intermingle with the corona radiata; ventral terminations are lateral to the corticospinal tract. AF, arcuate fasciculus; FAT, frontal aslant tract; FST, frontostriatal tract; IFOF, inferior fronto-occipital fasciculus; ILF, inferior longitudinal fasciculus; MLF, middle longitudinal fasciculus; SLF I, superior longitudinal fasciculus I; SLF II, superior longitudinal fasciculus II; SLF III, superior longitudinal fasciculus III; SLF-tp, temporoparietal component of the superior longitudinal fasciculus; UF, uncinate fasciculus.

Spatial relationship	Depth	Suprasylvian	Infrasylvian	Retrosylvian
Lateral	Superficial	SLF III, SLF II	ILF, AF	SLF-tp, SLF III, SLF II
	Middle	AF	MLF	AF, ILF
	Deep	IFOF, FAT^†^, FST^†^	UF, IFOF	MLF, IFOF
Reference structure	-	Corona radiata	Internal capsule	Inferior thalamic peduncle (optic and acoustic radiations)
Medial	-	SLF I, cingulum	-	-

**Video 1:** Three-dimensional representation of language-related white matter tracts and associated language disturbances during subcortical direct electrical stimulation. This 3D model illustrates the spatial organization of the major white matter tracts involved in language processing, displayed in relation to the cortical surface and ventricular system. Both dorsal and ventral stream pathways are represented, highlighting their anatomical course and spatial convergence within the perisylvian region. For each tract, typical language disturbances observed during positive intraoperative mapping are reported, providing a direct anatomo-functional correlation relevant for surgical planning and intraoperative decision-making. Courtesy of the Skull Base and Cerebrovascular Laboratory, University of California, San Francisco; reproduced with permission. CST, corticospinal tract; dAF, dorsal arcuate fasciculus; FAT, frontal aslant tract; FST, frontostriatal tract; IFOF, inferior fronto-occipital fasciculus; ILF, inferior longitudinal fasciculus; MLF, middle longitudinal fasciculus; SLF I, superior longitudinal fasciculus I; SLF II, superior longitudinal fasciculus II; SLF III, superior longitudinal fasciculus III; SLF-tp, temporoparietal component of the superior longitudinal fasciculus; UF, uncinate fasciculus; vAF, ventral arcuate fasciculus.

First, the SF serves as the principal anatomic landmark, dividing language-related tracts into three compartments: suprasylvian, infrasylvian, and retrosylvian.

Second, within each compartment, white matter bundles are characterized by their relationship to the corona radiata (and its caudal continuations, the internal capsule and inferior thalamic peduncle) as either lateral or medial.

Third, lateral tracts are stratified into three layers from superficial to deep (i.e., superficial, middle, and deep layers), encompassing exclusively association fibers organized according to their spatial position relative to the corona radiata. Medially, the corona is flanked by association fibers and commissural systems, with the frontal aslant tract (FAT) and frontostriatal tract (FST) occupying an intermediate position within the SMA. The lateral portion of FAT lies within the deep lateral layer, while the lateral portion of FST and the medial portions of both (i.e., FAT and FST) intermingle with the corona radiata and extend toward commissural territories. For the purpose of this surgical framework, FAT and FST are classified within the lateral deep layer (i.e., lateral to the corticospinal tract), acknowledging that their medial extensions cross anatomical boundaries.

This creates a compartmental 3 x 2 x 3 grid that localizes each tract by its (1) Sylvian compartment, (2) relationship to the corona radiata, and (3) depth layer, mirroring the conventional intraoperative navigation from surface to depth.

The 3D arrangement of the gyri and white matter structures, particularly the long association fibers, shows that their spatial organization mirrors the cranial contours superficially and the ventricular configuration in the deeper layers. For instance, the most prominent lateral point, the Euryon, overlies the most superficial and intricate network of fibers, which in turn extend inward toward the lateral ventricular wall and ultimately to the atrium.

Although both the anatomical organization and functional role of language-related white matter tracts must be interpreted within a dynamic hodotopical framework, the level of evidence supporting each tract-function relationship is not uniform and remains debated for some pathways. The anatomical course of most major association fibers is supported by a long tradition of white matter dissection studies and has been further refined by diffusion tractography. By contrast, functional interpretation relies on different levels of evidence, ranging from direct intraoperative observations during DES to indirect evidence from lesion studies, tractography, and functional imaging. Tracts such as the arcuate fasciculus (AF), superior longitudinal fascicle III (SLF III), inferior fronto-occipital fasciculus (IFOF), and FAT have relatively well-established anatomo-functional correlations in language mapping, whereas the language-related functional roles of the other pathways remain more variable, indirect, or context-dependent. Therefore, in the following sections, tract-function associations are presented according to the strength and nature of the available evidence, avoiding definitive functional assignments where the literature remains debated.

Anatomical and functional descriptions of the cingulate gyrus and cingulate fibers, thalamus, and basal ganglia, which are sometimes related to aspects of language, are beyond the scope of this article, as these structures are not conventionally mapped during awake surgery.

Short Association Fibers

**Figure 7:**
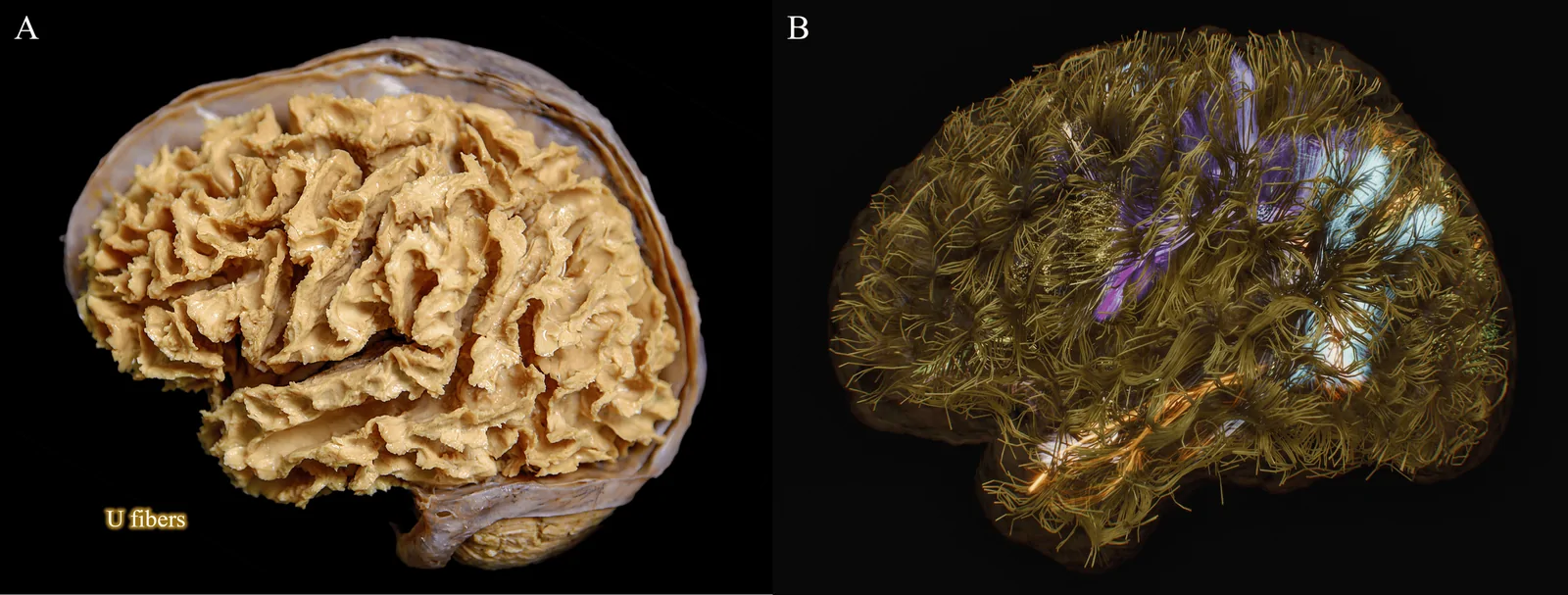
Short association fibers (U-fibers). (A) Lateral view of the left hemisphere showing a cadaveric dissection of short association fibers (U-fibers), connecting adjacent gyri along the cortical surface. (B) Tractographic reconstruction of superficial white matter pathways. U-fibers are widely distributed beneath the cortex. At a slightly deeper level, the most superficial portions of the long association fibers are appreciable, including the middle longitudinal fasciculus (orange), superior longitudinal fasciculus III (violet), superior longitudinal fasciculus II (purple), and the temporo-parietal component of the superior longitudinal fasciculus (light blue). Frontal and occipital terminations of the inferior fronto-occipital fasciculus (light green) are also visible. Courtesy of the Skull Base and Cerebrovascular Laboratory, University of California, San Francisco; reproduced with permission.

Short association fibers (SAFs), or U-fibers, represent up to 60% of total white matter volume, compared to about 10% for long association fibers (Figure 7). They are short (<30-50 mm), located superficially near the cortex, and often follow a U-shaped trajectory between adjacent gyri [10].

SAFs are crucial for brain connectivity but also highly vulnerable. Their delayed myelination, extending into adulthood, increases susceptibility to aging and disease. They also show higher vascularization and interstitial neuron density than deep white matter, reflecting their integrative role [10].

In language, SAFs contribute to early auditory processing, short-term memory, and phonemic retrieval, supporting the integration of auditory input into larger language networks [11].

Superior Longitudinal Fasciculus (SLF)

The SLF was first described in the nineteenth century by Reil and Autenrieth as a bundle of white matter fibers located in the temporal, parietal, and frontal regions, surrounding the SF. A century later, Petrides and Pandya identified three distinct segments of the SLF (SLF I, SLF II, and SLF III) and distinguished the SLF from the AF as two separate entities in the rhesus monkey. In humans, Makris was the first to segment the SLF, describing the three segments, plus the SLF IV, which corresponds to the AF [5].

**Figure 8:**
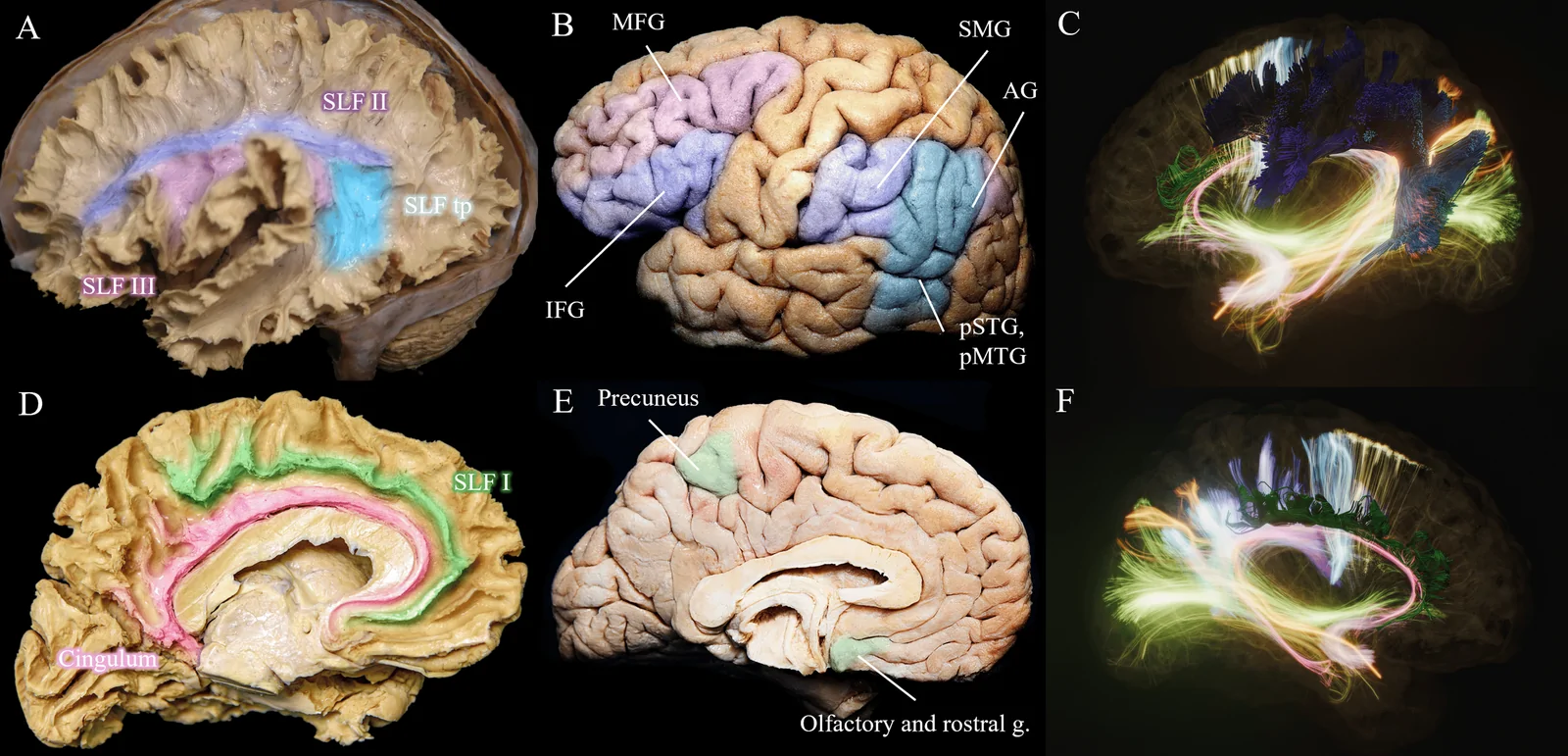
Superior longitudinal fasciculus. (A) Lateral view of the left hemisphere showing a cadaveric dissection of the SLF system. The different components can be appreciated: the SLF II running dorsally within the centrum semiovale, the SLF III in a more ventral and lateral position, and the SLF-tp, vertically oriented at the temporoparietal junction. (B) Lateral cortical view illustrating the main cortical terminations of the SLF system. The SLF II connects the AG with the MFG. The SLF III links the SMG with the IFG. The SLF-tp projects between the posterior temporal cortex (pSTG and pMTG) and inferior parietal regions. (C) Tractographic reconstruction of the lateral SLF system. The SLF II (dark blue) can be appreciated coursing within the centrum semiovale, connecting the AG with the MFG. The SLF III (purple) is seen more laterally, running between the SMG and the frontal operculum. The SLF-tp (light blue) shows a vertical orientation at the temporoparietal junction, linking the posterior temporal and inferior parietal cortices. (D) Medial view of a cadaveric dissection highlighting the most dorsomedial component of the system. The cingulum is visible, with the SLF I running superior to it along the medial superior frontal and parietal regions. (E) Medial cortical view showing the cortical terminations of the SLF I. This tract connects the precuneus with the medial superior frontal gyrus, including the olfactory and rostral gyri. (F) Tractographic reconstruction of the medial SLF system. The SLF I (green) can be appreciated as a dorsomedial pathway connecting the precuneus with the medial frontal regions, running above the cingulum (pink). Courtesy of the Skull Base and Cerebrovascular Laboratory, University of California, San Francisco; reproduced with permission. AG, angular gyrus; IFG, inferior frontal gyrus; MFG, middle frontal gyrus; pMTG, posterior middle temporal gyrus; pSTG, posterior superior temporal gyrus; SLF I–III, superior longitudinal fasciculus I–III; SLF-tp, temporoparietal component of the superior longitudinal fasciculus; SMG, supramarginal gyrus.

Today, most authors agree that the AF is anatomically and functionally distinct from the SLF, as the SLF is primarily a fronto-parietal system, while the AF is a fronto-temporal tract that traverses the parietal white matter beneath the SLF (Figure 8, Interactive Model 2) [12].

**Video 2:** Stepwise dissection of the white matter tracts involved in language, highlighting the FAT and its relationship with the SLF. This model illustrates a progressive virtual dissection of language-related white matter tracts. In the first stage, the FAT is isolated, highlighting its intersection with the SLF II at the level of the MFG. In the second stage, overlying cortical regions, including the MFG, SMG, temporoparietal junction, and AG, are removed, allowing clear exposure of the SLF III. In the third stage, further removal of the dorsolateral occipital cortex, MTG, and inferior temporal gyrus exposes the underlying white matter architecture, including the SLF-tp and ILF. Courtesy of the Skull Base and Cerebrovascular Laboratory, University of California, San Francisco; reproduced with permission. AG, angular gyrus; FAT, frontal aslant tract; FST, frontostriatal tract; IFG, inferior frontal gyrus; ILF, inferior longitudinal fasciculus; MFG, middle frontal gyrus; MTG, middle temporal gyrus; SFG, superior frontal gyrus; SLF II, superior longitudinal fasciculus II; SLF III, superior longitudinal fasciculus III; SLF-tp, temporoparietal component of the superior longitudinal fasciculus; SMA, supplementary motor area; SMG, supramarginal gyrus; STG, superior temporal gyrus.

SLF components are here described according to a topographic order, following a dorso-ventral and anterior-posterior distribution.

SLF I, the most dorsomedial component, connects the precuneus (medial superior parietal lobe) to the medial superior frontal gyrus (olfactory and rostral gyri), coursing above the cingulum and medial to the FAT and to the callosal fibers. Some anatomical descriptions place its trajectory through the corona radiata and the superolateral corpus callosum projections, suggesting a highly dorsal course. We are considering it medial to the corona radiata and in the suprasylvian compartment. It connects with motor and premotor areas (SMA complex) and establishes links between the precuneus and the anterior cingulate cortex. SLF I has been associated with the higher-level control of actions referenced to the body and with motor initiation and is not typically considered language-related. However, its classification as a distinct tract is not clearly defined, with some authors arguing that it may not be distinguishable from the cingulum due to their close anatomical relationship [12].

The SLF II connects the AG with the middle frontal gyrus (MFG). It runs through the centrum semiovale core, over the insula, and terminates in the posterolateral prefrontal region. It is still located in the superficial layer of the suprasylvian and retrosylvian lateral compartments, medial and superior to the SLF III. It courses near the superior margin of the atrium and along the body of the lateral ventricle, just underneath the IFS, crossing paths with the FAT and then curving to reach the AG and the middle occipital gyrus. Functionally, SLF II is involved in spatial and visuospatial awareness and attention, and lesions may result in neglect syndrome. Although not clearly defined, its connectivity profile also suggests a possible role in speech-related processes [12].

The most lateral segment, SLF III, connects the supramarginal gyrus (SMG) with the pars triangularis and opercularis of IFG, coursing through the IFG and frontoparietal operculum. This tract is located in the most superficial layer, both in its suprasylvian and retrosylvian lateral compartments. Along its path, it gives off projections to the frontal and parietal opercula, forming a roof over the anterior insula. The anatomical overlap between the anterior segment of AF and SLF III will be discussed further. In the dominant hemisphere, SLF III is associated with articulatory functions, including phonological processing, phono-motor integration, and verbal working memory. In the nondominant hemisphere, it appears to contribute to visuospatial attention, prosody, and aspects of music processing [13].

The temporoparietal SLF (SLF-tp), also referred to as the vertical SLF (vSLF), posterior segment of the arcuate fascicle (pAF), vertical AF (VAF), or temporo-parietal aslant tract (TPAT), has been described as an “extended U-fiber” with a compact vertical stem and divergent temporal and parietal terminations. Its central stem runs vertically between the temporal and parietal lobes, roughly at the level of the SF. It is located in the most superficial layer of the retrosylvian lateral compartment. Because of its cranio-caudal trajectory and departure from the longitudinal plane, this fasciculus can be regarded as a distinct subcomponent of the SLF. Some authors propose that it constitutes an independent fiber system, distinct from both SLF and AF [14]. A subdivision into ventral and dorsal tracts has been described: the ventral tract connects the dorsal and ventral aspects of the SMG, while the dorsal tract is connected to the AG. Both of them terminate in the posterior MTG and sometimes in the ITG. SLF-tp is mainly implicated in phonological processing.

Arcuate Fasciculus

**Figure 9:**
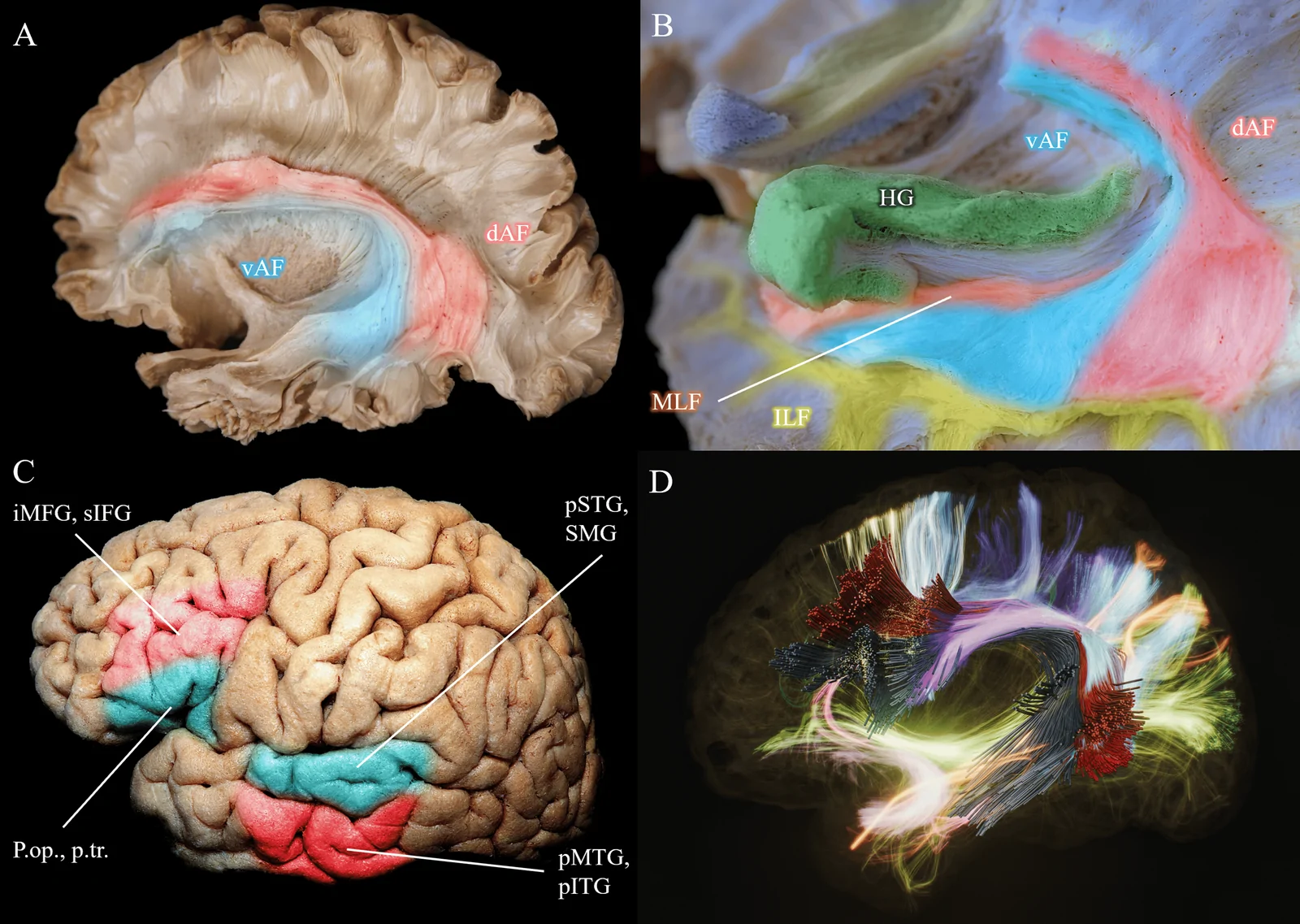
Arcuate fasciculus. (A) Lateral view of the left hemisphere showing a cadaveric dissection of the AF. The vAF and dAF can be appreciated, forming the characteristic C-shaped trajectory around the Sylvian fissure. (B) Magnified view of the temporoparietal junction. The vAF is seen coursing toward the pSTG, in close relationship with the MLF and ILF and medial to the more lateral opercular cortex, including the HG. The dAF is identified more dorsally. (C) Lateral cortical view illustrating the main cortical terminations of the AF. The dAF connects the iMFG and sIFG region with the pMTG and pITG. The vAF projects from the pars opercularis and ventral premotor cortex toward the pSTG and pMTG, passing through the inferior parietal region. (D) Tractographic reconstruction of the AF. The AF can be appreciated as a C-shaped perisylvian bundle connecting the frontal and posterior temporal regions. The vAF (blue) courses more inferiorly and medially, in close relationship with the MLF, whereas the dAF (red) runs more superiorly, passing just ventral to the SLF II. Courtesy of the Skull Base and Cerebrovascular Laboratory, University of California, San Francisco; reproduced with permission. AF, arcuate fasciculus; dAF, dorsal arcuate fasciculus; HG, Heschl’s gyrus; iMFG, inferior portion of the middle frontal gyrus; ILF, inferior longitudinal fasciculus; MLF, middle longitudinal fasciculus; pITG, posterior inferior temporal gyrus; pMTG, posterior middle temporal gyrus; pSTG, posterior superior temporal gyrus; sIFG, superior portion of the inferior frontal gyrus; SLF II, superior longitudinal fasciculus II; vAF, ventral arcuate fasciculus.

The AF, or direct segment of the AF, or long segment of the AF, is one of the most well-known associative fiber tracts, mainly due to its role in the classical language model. First described by Karl Burdach, it was historically considered a component of the SLF for a long time [5]. As previously noted, the AF is now understood as a fronto-temporal fasciculus, while the SLF primarily comprises fronto-parietal fibers (Figure 9, Interactive Model 3).

**Video 3:** Ventral and dorsal white matter pathways: anatomical relationships of the IFOF, UF, and AF. The first model focuses on ventral stream pathways, highlighting the IFOF and UF. Their spatial relationships with the sagittal stratum are demonstrated within the retrosylvian compartment. In particular, the IFOF, together with the MLF and the ILF, forms the more superficial component of the sagittal stratum, corresponding to its horizontal fibers. Deeper to these, the vertical fibers of the tapetum can be appreciated. The AF is visible in the frontal region, and then it is partially sectioned at the level of the supramarginal gyrus to expose the underlying sagittal stratum. The corona radiata is also sectioned, allowing visualization of the putamen in depth. The second model focuses on the AF, showing both its dorsal and ventral components as they course around the Sylvian fissure. The corona radiata is interrupted to reveal the underlying putamen. The MLF can be appreciated in relation to the vAF. Anteriorly, the temporal stem is exposed, with the IFOF and UF visible within it. Courtesy of the Skull Base and Cerebrovascular Laboratory, University of California, San Francisco; reproduced with permission. dAF, dorsal arcuate fasciculus; IFOF, inferior fronto-occipital fasciculus; ILF, inferior longitudinal fasciculus; MLF, middle longitudinal fasciculus; SMA, supplementary motor area; UF, uncinate fasciculus; vAF, ventral arcuate fasciculus.

Anatomically, the AF is described as a C-shaped perisylvian bundle connecting the pars opercularis and triangularis of the IFG with the posterior temporal cortex, including the posterior STS, STG, MTG, and potentially extending to the ITG. Along its path, it crosses the suprasylvian and retrosylvian lateral compartments, which are part of the middle layer, and then proceeds to the infrasylvian lateral compartment in the superficial layer.

Recently, two segments have been described [13]. The ventral segment originates from the pars opercularis and ventral premotor cortex, passes through the lower part of the SMG, and reaches the posterior STG and MTG. It courses very close to auditory radiations and middle longitudinal fasciculus (MLF), and it lies medial to SLF III. Functionally, it is strongly implicated in phonological processing; a lesion in this segment can result in phonemic paraphasia and repetition disorder, without semantic paraphasia, characteristic of conduction aphasia.

The dorsal segment runs just ventral to SLF II, passing through the inferior AG and connecting the inferior MFG with the posterior MTG and ITG. It is associated with lexical and semantic processing, as well as prosody. Damage to this segment has been linked to transcortical motor aphasia, characterized by impaired fluency with intact comprehension and repetition.

The AF specifically exhibits a strong dominant lateralization in both its connectivity and volume.

Frontal Aslant and Frontostriatal Tracts (FAT and FST)

**Figure 10:**
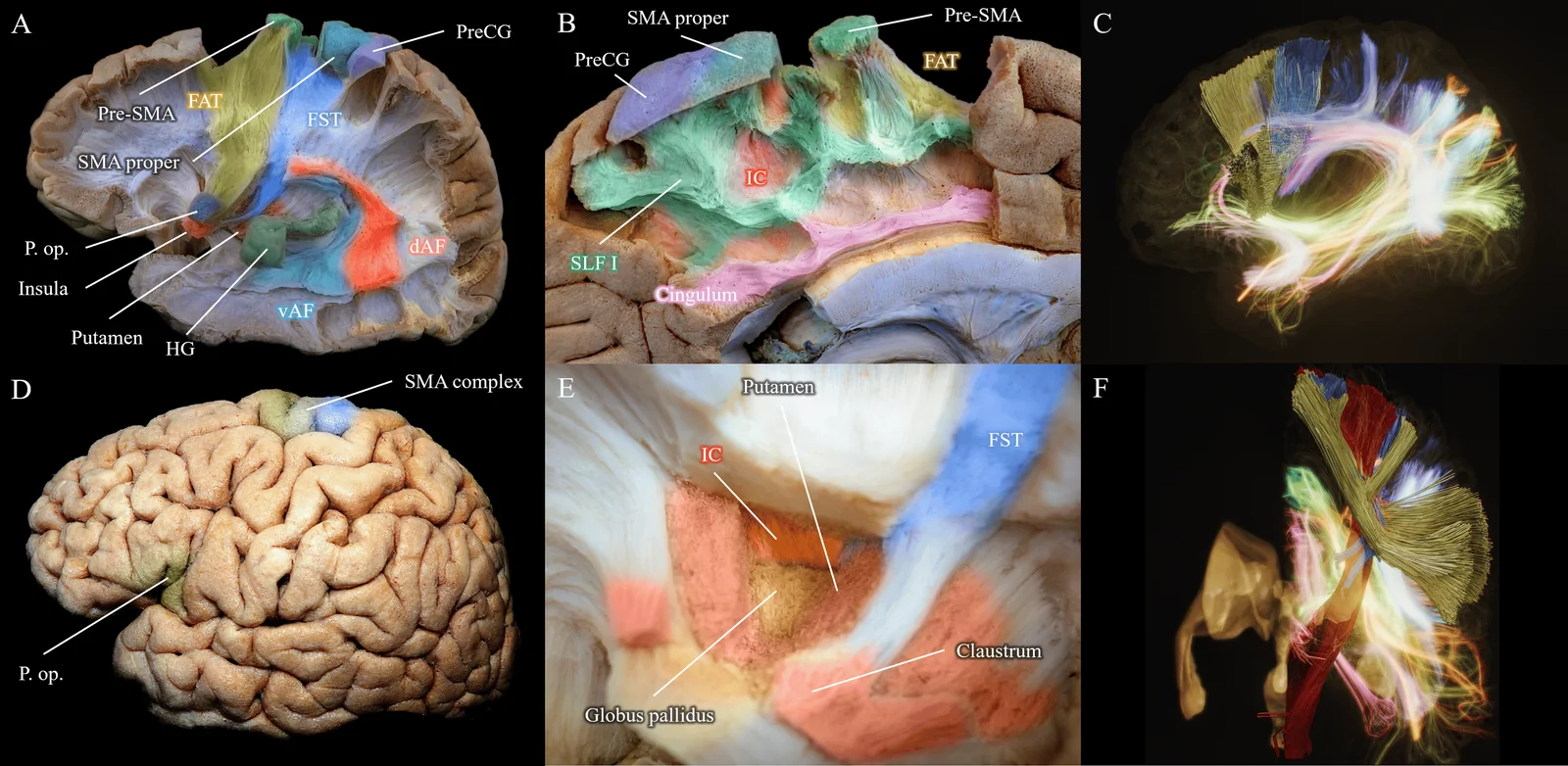
FAT and FST. (A) Lateral view of the left hemisphere showing a cadaveric dissection of the FAT and FST. The FAT can be appreciated arising from the SMA complex and coursing obliquely toward the pars opercularis. The FST is identified deeper, descending from the SMA complex toward the basal ganglia. The dAF and vAF are also visible in the perisylvian region. (B) Medial view highlighting the upper termination of the FAT. At the SMA level, fibers of the corona radiata and FAT are intermingled. From this region, the FAT courses laterally toward the inferior frontal gyrus. The internal capsule (IC), SLF I, and cingulum are also appreciable. (C) Tractographic reconstruction illustrating the spatial relationship between FAT and FST, lateral view. The FAT (yellow) can be appreciated as an oblique pathway connecting the SMA complex with the inferior frontal gyrus. The FST (light blue) descends toward the basal ganglia, running lateral to the internal capsule and adjacent to the corona radiata. (D) Lateral cortical view showing the cortical origins and terminations of the FAT. The tract connects the SMA complex with the pars opercularis and adjacent frontal regions. The deep termination of the FST is not visible in this view, as it projects toward the head of the caudate nucleus and the putamen. (E) Close-up of the deep structures illustrating the trajectory of the FST. The tract descends toward the basal ganglia, in close relationship with the internal capsule, claustrum, putamen, and globus pallidus. (F) Tractographic reconstruction of the frontal lobe associative pathways, anterior view. The FAT (yellow) and FST (light blue) are appreciable, with the FAT projecting laterally toward the frontal operculum, while the FST descends and curves medially to reach the basal ganglia. The IC (red) is also visible. Courtesy of the Skull Base and Cerebrovascular Laboratory, University of California, San Francisco; reproduced with permission. dAF, dorsal arcuate fasciculus; FAT, frontal aslant tract; FST, frontostriatal tract; HG, Heschl’s gyrus; IC, internal capsule; P. op., pars opercularis; PreCG, precentral gyrus; pre-SMA, pre-supplementary motor area; SLF I, superior longitudinal fasciculus I; SMA, supplementary motor area; vAF, ventral arcuate fasciculus.

The FAT is a relatively recently characterized white matter pathway. The connection between the pre-SMA and the IFG was first described by Aron et al. in 2007 using DTI. Later, in 2012, Catani and Thiebault de Scotten described the FAT through anatomical white matter dissection and formally named it, referencing its oblique (aslant) trajectory in the coronal plane (Figure 10, Interactive Models 2, 4). The FAT links the pre-SMA and the anterior SMA proper to the pars opercularis of the IFG. It runs medial to SLF II and superficial to the superior insular limiting sulcus. Along its course, it also forms connections with the dorsolateral prefrontal cortex (part of the SFG) and the anterior insula; additionally, some FAT fibers may extend to the inferior region of the precentral gyrus [15]. Furthermore, non-homologous callosal connections have been described between premotor areas across hemispheres: on this basis, the concept of a “crossed FAT” has been proposed, suggesting a possible contribution to functional recovery after SMA syndrome. FAT is located in the deep layer of the suprasylvian lateral compartment, although, to be precise, in the superior part, it is intermingled with CR.

**Video 4:** Stepwise dissection of ventral white matter pathways highlighting the ILF, MLF and IFOF. This model illustrates a progressive virtual dissection of white matter pathways. In the first stage, the frontal and parietal opercula are removed, with preservation of the pars opercularis of the IFG, as well as the temporal operculum except for Heschl’s gyrus. This exposes the insula, including both its short and long gyri. At this level, the SLF III is partially sectioned, while the SLF-tp and the Geschwind’s territory are prominently visible. Superiorly, the intersection between the FAT and the SLF II can be appreciated. With the removal of the SLF II, III, and -tp, the FAT becomes fully exposed, together with the FST. In the retrosylvian region, both the dorsal and ventral components of the AF are identified, along with the MLF projecting toward the superior parietal lobule. Inferior to the Sylvian fissure, the ILF is clearly visualized. Finally, after removal of the AF, the MLF is fully exposed. On the medial surface, the cingulum and SLF I can be appreciated, completing the depiction of the multilayered white matter architecture. Courtesy of the Skull Base and Cerebrovascular Laboratory, University of California, San Francisco; reproduced with permission. dAF, dorsal arcuate fasciculus; FAT, frontal aslant tract; FST, frontostriatal tract; IFG, inferior frontal gyrus; ILF, inferior longitudinal fasciculus; MLF, middle longitudinal fasciculus; SLF I, superior longitudinal fasciculus I; SLF II, superior longitudinal fasciculus II; SLF III, superior longitudinal fasciculus III; SLF-tp, temporoparietal component of the superior longitudinal fasciculus; SMA, supplementary motor area; vAF, ventral arcuate fasciculus.

Functionally, FAT is involved in speech initiation and verbal fluency. The SMA is often considered the premotor cortex of language, and the FAT is thought to be crucial for initiating speech output. Additional roles have been reported in lexical retrieval, grammatical processing, working memory, attention, and even music processing.

The FST, also known as the subcallosal fasciculus, links the pre-SMA and SMA proper to the basal ganglia, particularly the putamen and the head of the caudate nucleus, and to the insula. It runs posterior (in its superior portion) and medial (in its inferior portion) to the FAT, in the deep layer of the suprasylvian lateral compartment, descending beneath the superior insular limiting sulcus toward the central core of the brain. Fibers going to the lateral surface of the putamen, called the corticoputaminal tract, course along the lateral aspect of the internal capsule and reach the lateral surface of the putamen. In the SMA, the fibers of the corona radiata, FAT, and FST are intermingled; while descending, the FAT goes lateral, while the FST runs parallel and adjacent to the corona radiata, in particular to the claustrocortical tract (the lateral part of CR).

The FST plays a key role in voluntary motor initiation, and some authors regard it as the "final common pathway" through which the language network supports the motor implementation of speech.

Inferior Longitudinal Fasciculus (ILF)

**Figure 11:**
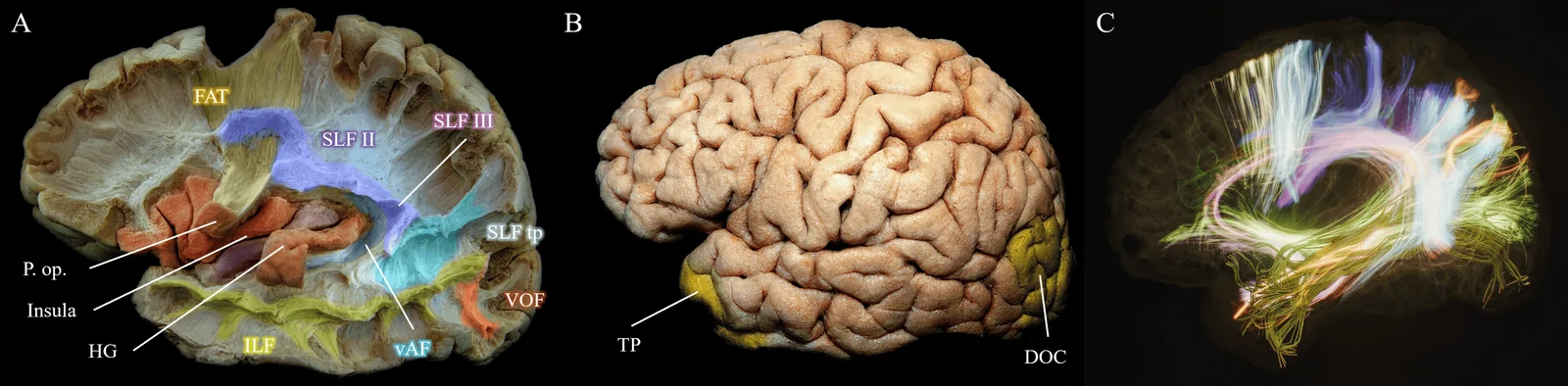
Inferior longitudinal fasciculus. (A) Lateral view of the left hemisphere showing a cadaveric dissection of the ILF. The tract is highlighted along the ventral temporal lobe, extending from the TP toward the occipital region. In the occipital lobe, the VOF is also appreciable. (B) Lateral cortical view illustrating the cortical terminations of the ILF. The tract links the TP with the DOC. (C) Tractographic reconstruction of the ILF. The ILF (yellow) is seen as a ventral pathway linking the TP to the occipital lobe, curving upward posteriorly toward the DOC. Courtesy of the Skull Base and Cerebrovascular Laboratory, University of California, San Francisco; reproduced with permission. DOC, dorsolateral occipital cortex; ILF, inferior longitudinal fasciculus; TP, temporal pole; VOF, ventral occipital fasciculus.

The ILF primarily links the temporal pole to the dorsolateral occipital cortex, without reaching the calcarine cortex (Figure 11, Interactive Model 4). As it approaches the preoccipital notch, it makes a distinct 45-degree upward bend to distribute its fibers to the dorsolateral occipital region. It courses just underneath the STS, running parallel to the SF, typically situated below and lateral to the temporal horn of the lateral ventricle. Along its path, the ILF establishes connections with MTG, ITG, the parahippocampal gyrus, the hippocampus, and the amygdala.

Spatially, the ILF lies lateral to the IFOF and the MLF. As it enters the occipital lobe, the ILF goes anterior to the vertical occipital fasciculus (involved in object identification), intersecting it around the occipital notch, and continues inferior and superficial to IFOF and MLF.

Thus, along its infrasylvian course, it is located in the superficial lateral compartment, while going retrosylvian, it occupies the middle lateral compartment.

The ILF belongs to the sagittal stratum (SS), a deep white matter complex of the temporo-parieto-occipital region, positioned below the inferior limiting sulcus of the insula and forming part of the lateral wall of the ventricular atrium. The SS is organized in three layers, from deep to superficial: the tapetum (fibers from the splenium of the corpus callosum, oriented vertically), the inferior thalamic peduncle (containing optic and acoustic radiations, oriented horizontally), and the association fibers (IFOF, MLF, and ILF). The ependyma of the lateral ventricle can be exposed by removing the tapetum [8, 13].

A primary function of the ILF is object identification and recognition, contributing to overall visual recognition. Furthermore, the ILF is considered a part of the ventral semantic pathway, contributing to language comprehension, alongside the MLF and extreme capsule; it also plays roles in reading and lexical retrieval [16].

Damage to areas traversed by ILF subcomponents, such as the left posterior fusiform gyrus, can impair high-spatial-frequency visual analysis, including both orthographic and non-orthographic processing, leading to pure alexia or anomia.

Despite its importance, resection involving the ITG and its underlying ILF has not consistently resulted in significant clinical deficits, even when performed in the dominant hemisphere, although specific, subtle language impairments cannot be excluded. This may reflect the brain’s functional redundancy and plasticity, especially in the context of slow-growing lesions.

Middle Longitudinal Fasciculus (MLF or MdLF)

**Figure 12:**
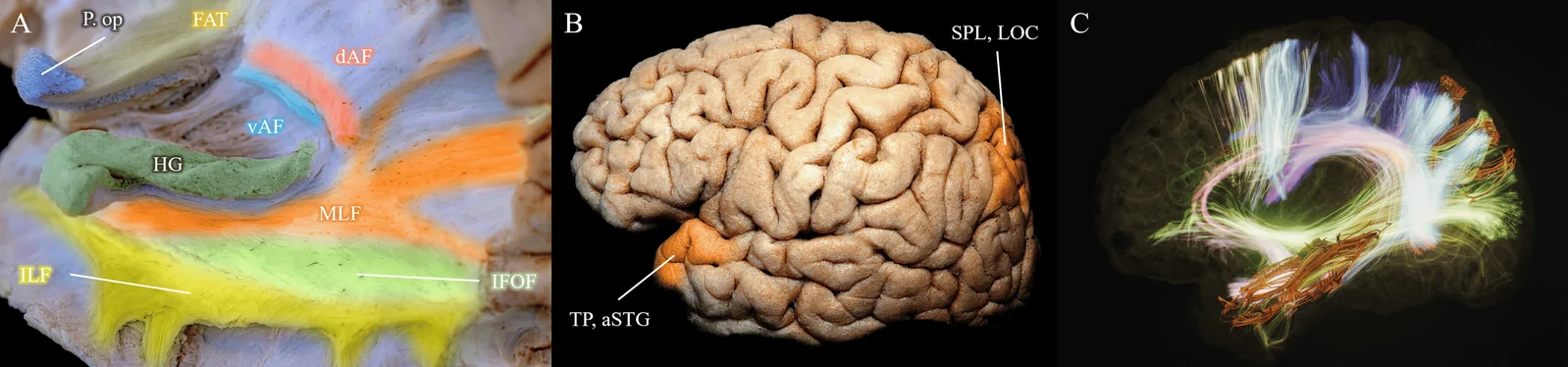
Middle longitudinal fasciculus. (A) Close-up view of the temporoparietal junction from a posterior perspective showing a cadaveric dissection of the MLF. The tract can be appreciated coursing within the superior temporal gyrus, in close relationship with the HG, and positioned medial and superior to the ILF and superficial to the IFOF. The vAF and dAF are also visible in the perisylvian region. (B) Lateral cortical view illustrating the cortical terminations of the MLF. The tract connects anterior temporal regions, including the TP and aSTG, with posterior parietal and occipital areas, including the SPL and LOC. Medial terminations toward the cuneus and precuneus are not visible in this view. (C) Tractographic reconstruction of the MLF. The MLF (orange) is seen as a longitudinal pathway extending from the anterior temporal regions toward the parietal and occipital cortices, with posterior projections toward the SPL, cuneus, precuneus, and LOC. Courtesy of the Skull Base and Cerebrovascular Laboratory, University of California, San Francisco; reproduced with permission. aSTG, anterior superior temporal gyrus; dAF, dorsal arcuate fasciculus; HG, Heschl’s gyrus; IFOF, inferior fronto-occipital fasciculus; ILF, inferior longitudinal fasciculus; LOC, lateral occipital cortex; MLF, middle longitudinal fasciculus; SPL, superior parietal lobule; TP, temporal pole; vAF, ventral arcuate fasciculus.

For many years, the MLF was relatively neglected and regarded as an enigmatic bundle, owing to the limited understanding of its morphology and function. The difficulty in establishing its functional role was underscored by the lack of conclusive data from DES during awake surgery or from lesion models following post-operative resections of the bundle. However, advances in high-angular-resolution DTI and microsurgical fiber dissection have more recently shed light on its anatomy and potential functions (Figure 12, Interactive Model 4).

The MLF primarily links the anterior STG with the occipital and parietal lobes, running through the STG, and is organized into two main segments [17]. The anteroventral segment (aMLF) links the temporal pole (TP) and planum polare (PP) to the superior and inferior portions of the lateral occipital cortex (LOC) and occipital pole; within the temporal region, it runs parallel, medial, and superior to the ILF. The posterior-dorsal segment (pMLF) connects the posterior STG, anterior transverse temporal gyrus (aTTG, or Heschl’s gyrus), and planum temporale (PT) to the SPL and superior LOC. The pMLF is more medial than the AF and the SLF-tp, intersects with the SLF-tp at the temporo-parietal junction, and runs superficial and superior to the acoustic radiation.

Parietal terminations of the MLF remain debated: while monkey studies and DTI in humans support projections to both the SPL and IPL, early human dissection studies reported terminations almost exclusively in the SPL, not in the IPL.

The MLF is part of the external layer of the sagittal stratum, along with the ILF and IFOF. At the level of the posterior insular point, the aMLF runs superficial to the IFOF, while the pMLF appears intermingled with it. MLF courses within the middle layer of the lateral infrasylvian compartment and then within the deep layer of the lateral retrosylvian compartment.

Functionally, the MLF is considered involved in higher-order processing of acoustic information. The pMLF, especially in the dominant hemisphere, contributes to the learning process associated with verbal-auditory stimuli. In contrast, the aMLF may be involved in retrieving and processing auditory information already consolidated in the temporal lobe [17].

Uncinate Fasciculus (UF) and Inferior Fronto-Occipital Fasciculus (IFOF)

**Figure 13:**
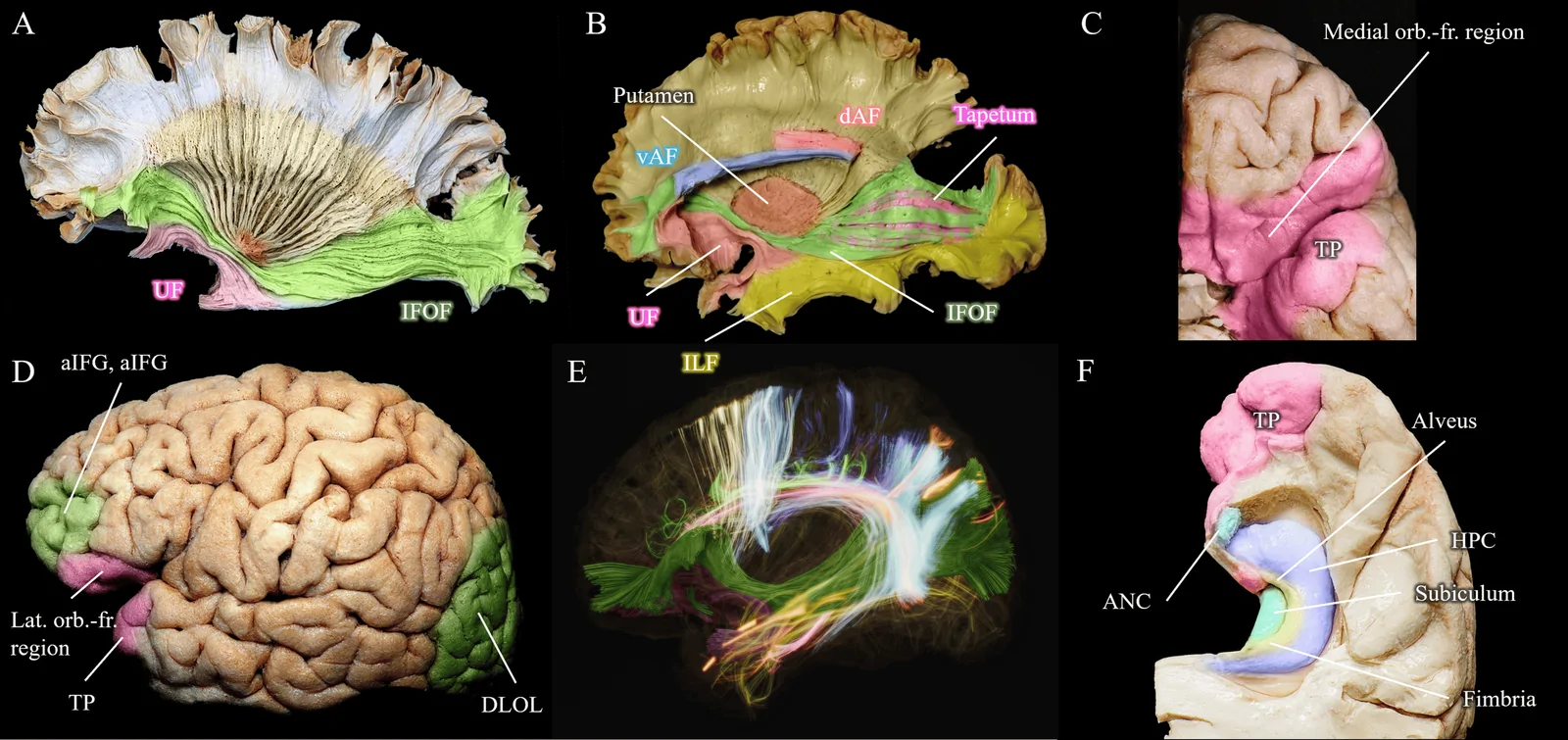
UF and IFOF. (A) Lateral view of the left hemisphere showing a cadaveric dissection of the UF and IFOF. The UF can be appreciated as a hook-shaped bundle connecting the anterior temporal lobe with the inferior frontal regions, whereas the IFOF is seen as a long anteroposterior pathway extending from the frontal to the occipital cortex. (B) Lateral view highlighting the deep course of the UF and IFOF. Part of the corona radiata has been removed to expose the putamen. In the retrosylvian region, the vertical fibers of the tapetum can be appreciated slightly deeper than the IFOF. The vAF and dAF are also visible. (C) Inferior view illustrating the medial frontal terminations of the UF, including the medial orbitofrontal cortex, septal area, and nucleus accumbens, whereas its temporal component converges toward the TP. (D) Lateral cortical view showing the lateral cortical projections. The UF links the TP with the lateral orbitofrontal region. The IFOF projects from the aIFG and extends posteriorly toward the DLOL. (E) Tractographic reconstruction of the ventral association pathways. The UF (pink) is appreciable as a hook-shaped bundle linking the anterior temporal and frontal regions, whereas the IFOF (green) appears as a long anteroposterior tract extending toward the posterior temporal, parietal, and occipital cortices. (F) Superior view of the temporal horn of the lateral ventricle (sectioned) and mesial temporal structures, such as the HPC and ANC. The temporal terminations of the UF can be appreciated, consisting of the TP, rhinal sulcus, parahippocampal gyrus, and semilunar gyrus. Courtesy of the Skull Base and Cerebrovascular Laboratory, University of California, San Francisco; reproduced with permission. aIFG, anterior inferior frontal gyrus; ANC, amygdaloid nuclear complex; dAF, dorsal arcuate fasciculus; DLOL, dorsolateral occipital lobe; HPC, hippocampus; IFOF, inferior fronto-occipital fasciculus; TP, temporal pole; UF, uncinate fasciculus; vAF, ventral arcuate fasciculus.

The UF is a hook-shaped fiber tract connecting the anterior temporal lobe to inferior frontal regions (Figure 13, Interactive Models 3, 4). More specifically, it links the temporal pole, uncus, and medial parahippocampus to the lateral orbitofrontal region (dorsolateral termination) and the medial orbitofrontal, nucleus accumbens, and septal areas (ventromedial termination). The UF courses in front of the anterior perforated substance, curves around the inferior and medial surface of the nucleus accumbens, and runs beneath the genu of the corpus callosum [13].

Within the subgenual region, the UF forms part of the ventral limbic pathway, complementing the cingulum, which is regarded as the dorsal limbic pathway. The UF lies near the ILF (ventral and superficial to the UF) and the IFOF (just dorsal to the UF) [13]. It is located in the deep layer of the infrasylvian lateral compartment.

The UF’s functional role remains debated. It is implicated in semantic processing, although clinical studies have reported variable outcomes following UF resection. Some evidence suggests that damage may impair proper name retrieval (“famous face naming”), hinting at a role in accessing consolidated, category-specific memories [1, 18]. Additionally, disconnection of the UF has been associated with behavioral disturbances, possibly due to disruption of limbic-frontal integration.

The IFOF, also called the fronto-occipital fasciculus (because a superior fronto-occipital fasciculus has not been described in humans), is a major anterior-posterior white matter bundle connecting widespread regions of the frontal, temporal, parietal, and occipital lobes (Figure 13, Interactive Models 3, 4). Its frontal terminations include the IFG (pars orbitalis and pars triangularis) and the dorsolateral prefrontal cortex (midpart of the MFG) [13]. From there, it passes deep to the anterior third of the superior limiting sulcus and beneath the upper half of the anterior limiting sulcus, forming the anterior floor of the external capsule. Running medially in the temporal lobe, it sends radiation to MTG and ITG, with terminations in the posterior temporal lobe and posterior parietal lobe and finally spreading and widening in the occipital lobe.

At the level of the limen insulae, the IFOF narrows, passing above the UF and then deep into the middle third of the inferior limiting sulcus. Its upper limit in the parieto-occipital region lies inferior to a reference line drawn from the midpoint of the limen insulae to the superior end of the parieto-occipital sulcus, which also marks the posterior edge of the claustrocortical fibers. From there, it continues posteriorly, terminating in the superior and middle temporal gyri and spreading into the occipital cortex.

IFOF is located in the deep layer of the three Sylvian (i.e., supra-, infra-, and retrosylvian) lateral compartments.

Functionally, the IFOF is strongly associated with semantic processing and represents a key anatomical substrate of the ventral semantic stream, enabling complex interlobar semantic integration. It has been implicated in meaningful speech comprehension and production, visual recognition, integration of multimodal sensory information, and motor planning; furthermore, it is thought to play an essential role in reading and writing processes. Finally, some sources suggest that the IFOF may participate in syntactic processing, and it is considered one of the ventral pathways involved in grammatical processing.

## Discussion

Contemporary language models: a work in progress

**Figure 14:**
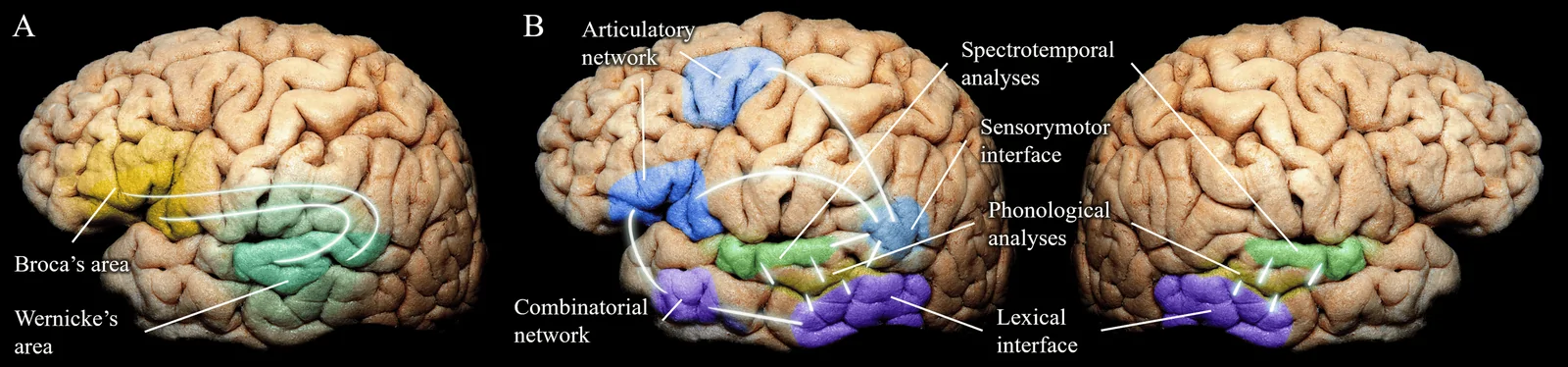
Classical model of language versus dual-stream model. (A) Left hemisphere cadaveric dissection illustrating the classical Wernicke–Geschwind model. Speech processing is depicted as a serial pathway: auditory input is processed in Wernicke’s area, located in the posterior superior temporal gyrus and supramarginal gyrus, and then relayed to Broca’s area, comprising the pars opercularis and pars triangularis of the inferior frontal gyrus, where motor speech representations are generated for articulation. (B) Schematic representation of the dual-stream model in both hemispheres. Speech processing begins in the superior temporal gyrus adjacent to Heschl’s gyrus, where acoustic input is transformed into phonemes. These are then conveyed to the middle temporal gyrus, where access to the lexicon and semantic processing occurs. Higher-level integration across sentences and context is supported by a distributed network involving temporal, inferior parietal, and frontal cortices, forming the combinatorial network. In parallel, phonological processing relies on interactions between posterior temporal and inferior parietal regions, converging at the sensorimotor interface in the Sylvian parietotemporal region, which translates auditory representations into motor commands. These are then executed by the precentral gyrus and dorsal premotor cortex for articulation. While early stages of auditory and semantic processing are largely bilateral, phonological processing and speech production show a predominant lateralization to the dominant hemisphere. Courtesy of the Skull Base and Cerebrovascular Laboratory, University of California, San Francisco; reproduced with permission.

The functional anatomy of speech has been the focus of extensive neuroscientific investigation for more than 130 years. In 1874, the classic Wernicke-Geschwind model was proposed [2]. Wernicke’s area is described in the posterior superior temporal gyrus (STG) and supramarginal gyrus (SMG) and, via the AF, is connected to Broca’s area, which is in the pars opercularis of the IFG. According to this model, a sensory word image is created in Wernicke’s area during speech perception, and, thanks to this connection, a motor word image would also emerge in Broca’s area (Figure 14A).

The traditional modular model of Broca’s and Wernicke’s areas, once revolutionary but now regarded as oversimplified, has been challenged by evidence showing that extensive resections of these regions do not necessarily result in permanent language deficits, suggesting that they function as nodes within a broader, adaptable network [2]. Advances in functional neuroimaging and DES during awake surgery have further revealed the brain’s remarkable plasticity and its ability to reallocate functions to alternative areas. Within this modern connectomic framework, language is understood as an emergent property of dynamic, distributed, and partially overlapping networks that include subcortical circuits and white matter pathways [1], thus shifting the paradigm from a static, modular view to a dynamic, hodotopical model.

The dual-stream model is currently the leading theory in the organization of language processing [3]. It proposes two parallel and interconnected pathways: the ventral stream, for speech recognition and semantic processing, and the dorsal stream, for phonological processing and speech production (Figure 14B).

The ventral stream supports speech recognition through spectrotemporal analysis in the posterior STG and Heschl’s gyrus, followed by phonological processing in the posterior STG and superior temporal sulcus (STS), which organizes sounds into phonemes. The involvement of not only the gyri but also the sulci is relevant within the dual-stream model, as the cortex must be considered in its complete three-dimensional continuity. Several eloquent regions, such as the superior temporal sulcus (STS) and Heschl’s gyrus (HG), lie deep within sulcal walls, making the sulci themselves critical components of functional language organization.

Through white matter connections, this information reaches the lexical interface in the posterior MTG and IFG, enabling access to semantic content. According to the Hickok-Poeppel model, this process is bilateral, whereas the Rauschecker-Scott model emphasizes hemispheric dominance. In the dominant hemisphere, the posterior MTG contributes to the combinatorial network and the anterior IFG to the articulatory network, enabling syntactic elaboration [1]. Overall, the ventral stream underlies speech comprehension by mapping sounds and visual input to meaning, supporting semantic representation, syntax, and lexical concepts, and by providing semantic content to the phonemic retrieval system during spontaneous speech. Key white matter tracts include the inferior fronto-occipital fasciculus (IFOF), inferior longitudinal fasciculus (ILF), and middle longitudinal fasciculus (MLF). The UF also mediates a frontotemporal network with a potential role in syntax, while the ILF plays a key role in lexical retrieval and reading. There are also connections with short-association fibers, or U-fibers, and adjacent gyri, especially around the posterior temporal lobe, Heschl’s gyrus, and inferior parietal lobule.

The dorsal stream is mainly responsible for sensorimotor integration, phonological processing, and mapping phonological information onto articulatory motor representations [1]. It plays a key role in speech development and normal speech production, including the acquisition and maintenance of basic articulatory-phonetic skills and the learning of new vocabulary. It is also involved in processing ordered sequences and sequence memory [4]. The sensorimotor interface, located in the Sylvian parietotemporal (Spt) area between the posterior STG and SMG, in the posterior planum temporale region just around the PSyP, translates cortical input into motor commands. Although not speech-specific, as it also responds to music, it is considered the vocal counterpart of visuomotor transformation regions. The Spt connects with the frontal lobe, particularly the IFG (pars triangularis and opercularis) and the posterior MFG (dlPMC), anterior to the precentral gyrus, which forms the core of the articulatory network. The dorsal stream is usually lateralized to the dominant hemisphere. Key white matter tracts connecting these areas include the SLF (particularly SLF II and SLF III), the temporoparietal SLF (SLF-tp), and the AF.

The SMA and its associated white matter pathways play a key role in the spontaneity and initiation of speech. These functions rely on the connection with the IFG through the FAT. SMA has specific motor functions, including intention and the initiation of motor activity. It has a somatotopical organization; the inferior limb, superior limb, and face are represented from posterior to anterior, respectively [9]. The pre-SMA has been implicated in several higher-order processes, including language production, movement recognition, action intention, memory storage, visuomotor integration, learning, time perception, conflict resolution, and switching between actions. SMA and pre-SMA have important connections with the contralateral SMA via short U-fibers, SLF I, the cingulate gyrus, the limbic system, claustrocortical fibers, callosal fibers, and the corticospinal tract (CST). The frontostriatal tract (FST) is strictly connected to the basal ganglia, and the FAT has connections with the insula. The SMA complex is associated with the SMA language syndrome, characterized by transient difficulty initiating speech despite preserved repetition, with gradual recovery usually occurring over months to years.

Finally, speech requires the coordinated activity of the laryngeal, pharyngeal, lingual, oral, and facial muscles, all of which have extensive cortical representation in the primary motor cortex of the posterior inferior precentral gyrus. Together with the pars opercularis of the IFG, the anterior precentral gyrus forms the ventral premotor cortex (vPMC) [5]. The left vPMC, particularly its lateral portion behind the pars opercularis, is regarded as the “final common pathway for speech" with limited plasticity, as its resection leads to permanent articulatory deficits [2].

Tables 3, 4 summarize the cortical and white matter structures involved in language according to the classical Wernicke-Geschwind and dual-stream models, comparing their functional roles and highlighting the conceptual evolution from a localizationist to a network-based framework [1,3,5,13,15-17].

**Table 3:** Comparison between the classical Wernicke-Geschwind model and the dual-stream model based on the involved cortical structures and their functions. IFG, inferior frontal gyrus; ITG, inferior temporal gyrus; MFG, middle frontal gyrus; MTG, middle temporal gyrus; SMG, supramarginal gyrus; SPL, superior parietal lobule; STG, superior temporal gyrus; STS, superior temporal sulcus. Sources: References [1,3,5].

Classical model		Dual-stream model	
Cortical area	Function	Cortical area	Function
–	–	Heschl's gyrus, posterior STG	Spectro-temporal analysis of speech sounds: registration of the sounds
–	–	Posterior STG, STS	Phonological analysis: ordering of the sounds into phonemes
–	–	Posterior MTG, IFG	Lexical interface: access semantic content and assume a meaning
–	–	Temporal pole	Combinatorial network
–	–	Anterior IFG	Articulatory network
Wernicke's area (i.e., posterior STG)	Sensory word image creation	Sylvian parietotemporal region (i.e., posterior STG, SMG)	Sensorimotor interface: cortical input is translated into motor commands
Broca's area (i.e., pars triangularis and opercularis in IFG)	Motor word image creation	Pars triangularis and opercularis in IFG	Phonological processing, articulation
–	–	Posterior MFG	Articulation

**Table 4:** Comparison between the classical Wernicke-Geschwind model and the dual-stream model based on the involved white matter structures, their terminations, and functions. AF, arcuate fasciculus; AG, angular gyrus; ant., anterior; FAT, frontal aslant tract; FST, frontostriatal tract; IFG, inferior frontal gyrus; IFOF, inferior fronto-occipital fasciculus; ILF, inferior longitudinal fasciculus; inf., inferior; ITG, inferior temporal gyrus; lat., lateral; med., medial; MFG, middle frontal gyrus; MLF, middle longitudinal fasciculus; MTG, middle temporal gyrus; post., posterior; op., opercularis; pre-SMA, pre-supplementary motor area; SLF I, superior longitudinal fasciculus I; SLF II, superior longitudinal fasciculus II; SLF III, superior longitudinal fasciculus III; SLF-tp, temporoparietal component of the superior longitudinal fasciculus; SMA, supplementary motor area; SMG, supramarginal gyrus; SPL, superior parietal lobule; STG, superior temporal gyrus; sup., superior; tri., triangularis; UF, uncinate fasciculus. Sources: References [1,3,5,13,15-17].

Classical model			Dual-stream model		
White matter tract	Terminations	Function	White matter tract	Terminations	Function
AF	Frontal: Broca's area (i.e., pars tri. and op. in IFG); Temporal: Wenicke's area (i.e., posterior STG)	Connection between sensory and motor areas	AF	Dorsal segment: Frontal (inf. MFG, sup. IFG); Temporal (post. ITG, MTG). Ventral segment: Frontal (pars op./tri.); Temporal (post. STG, MTG)	Dorsal segment: lexical and semantic processing, prosody. Ventral segment: phonological processing
–	–	–	SLF III	Frontal: pars tri. and op.; Parietal: SMG	Phonological processing, working memory
–	–	–	SLF II	Frontal: MFG; Parietal: AG	Regulation of spatial and visuospatial awareness and attention
–	–	–	SLF I	Frontal: olfactory/rostral gyrus (medial SFG); Parietal: precuneus, marginal sulcus	Higher-level regulation of body-centered actions and motor initiation
–	–	–	SLF-tp	Parietal: inf. parietal lobule; Temporal: post. STG and MTG	Phonological processing
–	–	–	FAT	Superior: pre-SMA/SMA proper; Inferior: pars opercularis	Verbal fluency, speech initiation, lexical retrieval, grammatical processing, attention, working memory, music processing
–	–	–	FST	Superior: SMA proper/pre-SMA; Inferior: basal ganglia, insula	Motor initiation, potential role in speech control
–	–	–	ILF	Temporal: temporal pole; Occipital: dorsolateral occipital cortex	Object identification, visual recognition, language comprehension, lexical retrieval, reading
–	–	–	MLF	Temporal: temporal pole, ant. STG; Parietal: SPL, precuneus; Occipital: lat. occipital/cuneus	Higher-order acoustic functions; verbal-auditory learning; auditory information retrieval
–	–	–	UF	Temporal: temporal pole, uncus, parahippocampus; Frontal: lat./med. orbitofrontal, nucleus accumbens	Semantic function, famous face naming
–	–	–	IFOF	Frontal: orbitofrontal, ant. MFG/IFG; Occipital: dorsolateral occipital lobe	Semantic processing, visual recognition, multimodal integration, motor planning, reading, writing, speech comprehension and production

Language mapping

During awake cortical mapping with DES, different types of errors can be elicited depending on the site of stimulation (Interactive Models 1, 5). Among all speech and language disturbances reported during DES, 59.0% occur at the cortical level and 40.4% at the subcortical level, highlighting the critical relevance of both compartments [18].

**Video 5:** Cortical gyri and associated language disturbances during cortical direct electrical stimulation. This model illustrates the cortical surface anatomy, highlighting key gyri involved in language function. Each cortical site is associated with the most commonly observed language disturbances during positive direct electrical stimulation. The model emphasizes the functional organization of both dorsal and ventral language networks at the cortical level, as well as the relationship between classical eloquent areas and surrounding regions frequently involved during intraoperative mapping. Key craniometric and surgical landmarks are also included to facilitate anatomical orientation. Courtesy of the Skull Base and Cerebrovascular Laboratory, University of California, San Francisco; reproduced with permission. ASyP, anterior Sylvian point; dlPMC, dorsolateral premotor cortex; IFG, inferior frontal gyrus; IRP, inferior Rolandic point; MTG, middle temporal gyrus; PSyP, posterior Sylvian point; SMA, supplementary motor area; SMG, supramarginal gyrus; SPL, superior parietal lobule; SRP, superior Rolandic point; STG, superior temporal gyrus.

It is essential to keep in mind that anatomy varies between individuals; pathology may distort standard architecture, and neuroplasticity can redistribute functions. Moreover, no function derives from a single brain structure but rather from the output of a computational network that links cortical regions through white matter pathways. Anatomical and functional boundaries rarely overlap perfectly, meaning that DES may evoke responses in unexpected locations or, conversely, may fail to elicit reactions in areas where they might traditionally be anticipated. As with cortical mapping, assuming that a single fiber tract subserves a single function is an oversimplification. White matter bundles generally support multiple, often overlapping functions, and it is the integrated activity of the entire network that enables complex behavior.

Therefore, a solid understanding of the anatomy and the key features of all relevant structures is essential during language mapping, not only to select the most appropriate tasks for each region but also to avoid “false friends," defined as potentially misleading responses during DES (e.g., a motor response mistaken for speech arrest) that may otherwise lead to an unwarranted and premature interruption of tumor debulking.

A detailed overview of the association between anatomical regions and the errors most commonly elicited during DES is presented in Table 5 (organized by region) and Table 6 (organized by error type) [1, 18, 19]. Although many responses show a clear preference for the dominant hemisphere, particularly within dorsal-stream structures, errors have also been reported in the non-dominant hemisphere. This bilaterality should not be considered a rule, but rather a possibility to account for during surgery.

**Table 5:** Most common errors associated with anatomical regions. dAF, dorsal arcuate fasciculus; FAT, frontal aslant tract; FST, frontostriatal tract; IFG, inferior frontal gyrus; IFOF, inferior fronto-occipital fasciculus; ILF, inferior longitudinal fasciculus; ITG, inferior temporal gyrus; MLF, middle longitudinal fasciculus; MTG, middle temporal gyrus; SLF I, superior longitudinal fasciculus I; SLF II, superior longitudinal fasciculus II; SLF III, superior longitudinal fasciculus III; SLF-tp, temporoparietal component of the superior longitudinal fasciculus; SMA, supplementary motor area; SMG, supramarginal gyrus; SPG, superior parietal gyrus; STG, superior temporal gyrus; UF, uncinate fasciculus; vAF, ventral arcuate fasciculus. Sources: References [1,18,19].

Cortical location	Side	Error
Dorsal stream		
Pre-central gyrus	Bilateral	Speech arrest, anomia.
	Dominant	Dysarthria/anarthria, production errors, phonemic errors, semantic errors, morphosyntactic errors, verbal apraxia.
Dorsolateral premotor cortex	Dominant	Anomia.
IFG (pars opercularis and triangularis)	Bilateral	Speech arrest, anomia; semantic paraphasias (mostly in pars triangularis).
	Dominant	Morphosyntactic errors, dysarthria/anarthria, phonemic errors, production errors.
SMG	Bilateral	Semantic errors, phonemic paraphasias, speech arrest.
	Dominant	Anomia, semantic errors, dysarthria/anarthria, production errors, comprehension errors.
SMA proper	Bilateral	Speech initiation difficulties.
Ventral stream		
Posterior STG	Bilateral	Semantic errors, anomia, comprehension errors.
	Dominant	Production errors, phonemic paraphasia.
MTG	Bilateral	Anomia, reading errors, semantic errors (rare).
	Dominant	Phonemic paraphasia, morphosyntactic errors.
ITG	Bilateral	Reading errors, anomia, semantic errors (rare).
Post-central gyrus	Bilateral	Speech arrest, verbal apraxia, dysarthria/anarthria.
SPG	Dominant	Writing errors.
Basal occipitotemporal cortex	Bilateral	Visual paraphasias.
Subcortical location	Side	Error
Dorsal stream		
SLF III	Bilateral	Dysarthria/anarthria, or repetition of articulatory errors.
SLF-tp	Bilateral	Phonemic repetition errors.
SLF II	Bilateral	Transient decrease in the appreciation of visual stimuli on the contralateral side.
SLF I	Bilateral	No language alterations.
vAF	Bilateral	Phonemic paraphasia, repetition disorder, anomia.
dAF	Bilateral	Impaired fluency.
FAT	Dominant	Speech initiation difficulties, reduction of speech fluency, speech arrests, stuttering, production errors, morphosyntactic errors, semantic errors, perseverations.
FST	Bilateral	Speech initiation difficulties with intact repetition, speech arrests.
	Dominant	Anomia.
Ventral stream		
ILF	Bilateral	No language disturbance, although reading errors, anomia without paraphasias are rarely reported.
	Dominant	Phonemic errors (rare).
MLF	Bilateral	Often, no language alterations, rarely phonemic errors, and anomia.
UF	Dominant	No language modifications; phonemic, production, and perseverations are rarely reported.
IFOF	Bilateral	Semantic errors, comprehension errors.
	Dominant	Anomia, perseverations, morphosyntactic errors, reading errors, writing errors.

**Table 6:** Most common eloquent anatomical regions associated with positive mapping. AF, arcuate fasciculus; dlPMC, dorsolateral premotor cortex; FAT, frontal aslant tract; FST, frontostriatal tract; IFG, inferior frontal gyrus; IFOF, inferior fronto-occipital fasciculus; ILF, inferior longitudinal fasciculus; IPL, inferior parietal lobule; ITG, inferior temporal gyrus; MTG, middle temporal gyrus; PreCG, precentral gyrus; SMA, supplementary motor area; SLF III, superior longitudinal fasciculus III; SMG, supramarginal gyrus; SPG, superior parietal gyrus; STG, superior temporal gyrus; UF, uncinate fasciculus; vPMC, ventral premotor cortex. Sources: References [1,18,19].

Type of Error	Practical Example	Diagnostic Task	Cortical Sites	Subcortical Sites
Speech arrest	Patient is asked to count from 1 to 20, but suddenly he cannot produce any sound.	Picture naming, repetition, and counting.	vPMC, Precentral gyrus, IFG	SLF III, FAT, FST
Dysarthria/Anarthria	Patient says “table” but output is slurred or unintelligible.	Word repetition, spontaneous speech.	vPMC, PreCG	SLF III
Production errors	Difficulty initiating speech or constructing sentences; e.g., says “runner” instead of “to run”.	Spontaneous speech, narrative task.	IFG, PreCG, SMA, STG	FAT, FST
Morphosyntactic errors	Says “He goes to school” instead of “He goes to school”.	Sentence construction.	IFG, MTG	-
Phonemic paraphasia	Says “lorse” instead of “horse”.	Picture naming, repetition.	Posterior STG, IFG, MTG, SMG	AF, SLF III
Anomia	Sees a chair, describes its use, but can’t retrieve the word.	Picture naming, object use description.	Posterior STG, MTG, ITG, dlPMC, IFG	IFOF, UF
Semantic paraphasia	Shown a dog, says “cat” or “animal”.	Picture naming, semantic association task.	Posterior MTG, anterior SMG, IFG, STG	IFOF, UF
Perseverations	Says “dog” repeatedly for “dog”, “cat”, and “house”.	Series naming, object naming.	—	IFOF
Reading errors	Misreads or cannot read “bottle” (pure alexia, phonological errors).	Word reading.	MTG, ITG, IPL	ILF, IFOF
Comprehension errors	Asked to point to the ceiling, but points to the floor.	Object identification, command following.	Frontal, temporal, and parietal lobes	IFOF
Speech initiation difficulty	Delayed or absent initiation of speech after a prompt.	Narrative description, open question.	SMA	FAT, FST
Writing errors	Difficulty writing words or phrases, illegible output.	Writing task.	Superior parietal gyrus (SPG)	IFOF
Verbal apraxia	Inconsistent articulation errors, effortful attempts (e.g., “bo-tone”, “boh-ttone”).	Repetition, sound articulation tasks.	Postcentral gyrus	SLF III
Visual paraphasias	Sees a banana and says, “yellow curved thing”.	Picture naming, object feature description.	Basal occipitotemporal cortex	—

Final considerations

This study offers, for the first time, a comprehensive overview of the anatomy underlying language function. It provides a more practical delineation of previously described cortical structures and white matter tracts, which, within a more up-to-date dynamic hodotopical framework, can be considered the main structures of computational neuroanatomy while establishing correlations among craniometric landmarks, corresponding cortical regions, and underlying subcortical pathways.

The compartmental classification presented in this manuscript is offered as a surgical navigational aid, not a biological claim. It organizes known anatomical structures into an operative sequence. Tracts such as the FAT and FST remind us that white matter bundles are complex volumetric entities that respect grids only approximately and that ongoing taxonomic debates (e.g., indirect segments of the AF) remain unresolved here. The asymmetric compartmental grid is a practical layover for an intricate set of circuits that will require adjustments as intraoperative mapping and connectomics advance.

The evolution of our understanding of brain organization, particularly with respect to language, illustrates how neuroscientific progress can reshape surgical practice. The transition from the classical model, grounded in rigid localizationism, to more sophisticated network models, such as the dual-stream model, reflects a shift towards the aforementioned hodotopical perspective, where multiple structures continuously interact, remodel, and dynamically process information.

Neurosurgeons have played a pivotal role in driving this conceptual change. As early as 1959, Penfield demonstrated that no single “speech production area” exists, opening the way for more extensive resections without long-term language consequences. The accumulated experience from patients undergoing DES during awake procedures has dismantled Broca’s myth and emphasized both neuroplasticity, particularly evident in low-grade gliomas, and the critical contribution of the entire white matter network. In this setting, intraoperative language mapping has become the gold standard for achieving a supramarginal resection, maximizing oncological outcomes while preserving long-term language function.

Surgical planning today reflects this paradigm shift, moving beyond technical execution to incorporate detailed anatomical and functional knowledge, allowing resections to be tailored to each patient’s priorities. In some cases, this may mean favoring the preservation of critical abilities, such as professional or artistic performance, over a maximally aggressive oncological resection.

Moreover, an “eloquent” brain is not limited to motor, sensory, and language systems. Large-scale cognitive and behavioral networks, including the default mode network (DMN), central executive network (CEN), salience network (SN), dorsal attention network (DAN), and ventral attention network (VAN), are increasingly recognized as essential to quality of life. Their disruption can lead to neuropsychiatric and cognitive syndromes such as depression, attentional deficits, memory disturbances, abulia, or akinetic mutism [20]. This broader view reinforces the need for neurosurgeons to integrate connectomics into preoperative planning and intraoperative mapping.

Emerging technologies, particularly artificial intelligence (AI) and machine learning, are beginning to influence connectomics by enabling advanced modeling of brain networks and supporting automatic, high-resolution white matter segmentation [2]. In the near future, these tools may allow real-time integration of functional connectivity into surgical navigation, enhancing decision-making, refining patient-specific risk stratification, and guiding targeted neurorehabilitation strategies that leverage plasticity and redundancy in brain networks.

## Conclusions

The present work offers a modern and detailed description of the 3D computational anatomy of the structures underlying language models. By organizing the perisylvian and perirolandic networks according to their Sylvian compartment, relationship to the corona radiata, and depth layer, this study provides a spatial ontology that mirrors the intraoperative trajectory from surface to depth. Microsurgical cadaver dissections and tractography, combined with a specific focus on DES data, form the core of this study, which aims to be exhaustive, practical, and accessible.

The current hodotopical and patient-centered view of cortico-subcortical surgery has greatly improved our understanding of inter- and intra-individual variability in the networks involved in language. However, it will be paramount for neurosurgeons and trainees to continuously follow the ever-evolving fields of neurolinguistics and computational anatomy, which will surely provide more granular models and a deeper understanding of human language and related functions in the years to come.

This work is intended as a structured, functional operative reference for neurosurgeons, fostering active, reciprocal collaboration with other professionals to continually expand the therapeutic options available to patients with brain tumors and their families.
